# Phytochemicals and Regulation of NF-kB in Inflammatory Bowel Diseases: An Overview of In Vitro and In Vivo Effects

**DOI:** 10.3390/metabo13010096

**Published:** 2023-01-07

**Authors:** Lucas Fornari Laurindo, Ana Rita de Oliveira dos Santos, Antonelly Cassio Alves de Carvalho, Marcelo Dib Bechara, Elen Landgraf Guiguer, Ricardo de Alvares Goulart, Renata Vargas Sinatora, Adriano Cressoni Araújo, Sandra Maria Barbalho

**Affiliations:** 1 Department of Biochemistry and Pharmacology, School of Medicine, University of Marília (UNIMAR), Avenida Hygino Muzzy Filho 1001, São Paulo 17525-902, Brazil; 2 Postgraduate Program in Structural and Functional Interactions in Rehabilitation, University of Marília (UNIMAR), Avenida Hygino Muzzy Filho 1001, São Paulo 17525-902, Brazil; 3 Department of Biochemistry and Nutrition, School of Food and Technology of Marília (FATEC), Avenida Castro Alves 62, São Paulo 17500-000, Brazil

**Keywords:** inflammatory bowel diseases, ulcerative colitis, Crohn’s disease, intestinal inflammation, phytochemicals, nuclear factor kappa B, NF-kB, inflammation, anti-inflammatory

## Abstract

Inflammatory bowel diseases (IBD) are chronic relapsing idiopathic inflammatory conditions affecting the gastrointestinal tract. They are mainly represented by two forms, ulcerative colitis (UC) and Crohn’s disease (CD). IBD can be associated with the activation of nuclear factors, such as nuclear factor-kB (NF-kB), leading to increased transcription of pro-inflammatory mediators that result in diarrhea, abdominal pain, bleeding, and many extra-intestinal manifestations. Phytochemicals can interfere with many inflammation targets, including NF-kB pathways. Thus, this review aimed to investigate the effects of different phytochemicals in the NF-kB pathways in vitro and in vivo models of IBD. Fifty-six phytochemicals were included in this study, such as curcumin, resveratrol, kaempferol, sesamol, pinocembrin, astragalin, oxyberberine, berberine hydrochloride, botulin, taxifolin, naringin, thymol, isobavachalcone, lancemaside A, aesculin, tetrandrine, Ginsenoside Rk3, mangiferin, diosgenin, theanine, tryptanthrin, lycopene, gyngerol, alantolactone, mangostin, ophiopogonin D, fisetin, sinomenine, piperine, oxymatrine, euphol, artesunate, galangin, and nobiletin. The main observed effects related to NF-kB pathways were reductions in tumor necrosis factor-alpha (TNF-α), interleukin (IL)-1β, IL-6, interferon-gamma (IFN-γ), and cyclooxygenase-2 (COX-2), and augmented occludin, claudin-1, zonula occludens-1, and IL-10 expression levels. Moreover, phytochemicals can improve weight loss, stool consistency, and rectal bleeding in IBD. Therefore, phytochemicals can constitute a powerful treatment option for IBD in humans.

## 1. Introduction

Inflammatory bowel diseases (IBD) are relapsing idiopathic and chronic inflammatory conditions affecting the gastrointestinal tract. They are mainly characterized by two entities named ulcerative colitis (UC), involving a continuous inflammatory process of the colonic mucosa, and Crohn’s disease (CD), which can produce ulceration lesions anywhere in the gastrointestinal tract. Both environmental and genetic factors are associated with disrupting the intestinal epithelial barrier, resulting in increased permeability and augmented uptake of microorganisms triggering the activation of an imbalanced immune response and leading to inflammation. Environmental factors can be related to diet, food additives, distress, infections, medications, reduced levels of vitamin D, and oxidative stress (OS). Regarding genetic factors, modifications in genes related to the expression of proteins and receptors of immune system cells can be associated with the activation of nuclear factors, such as nuclear Factor-kB (NF-kB). This activation leads to increased transcription of pro-inflammatory mediators, such as interleukin (IL)-1β, IL-6, tumor necrosis factor-α (TNF-α), and interferon (IFN)-γ [1,2,3,4].

**Figure 1:**
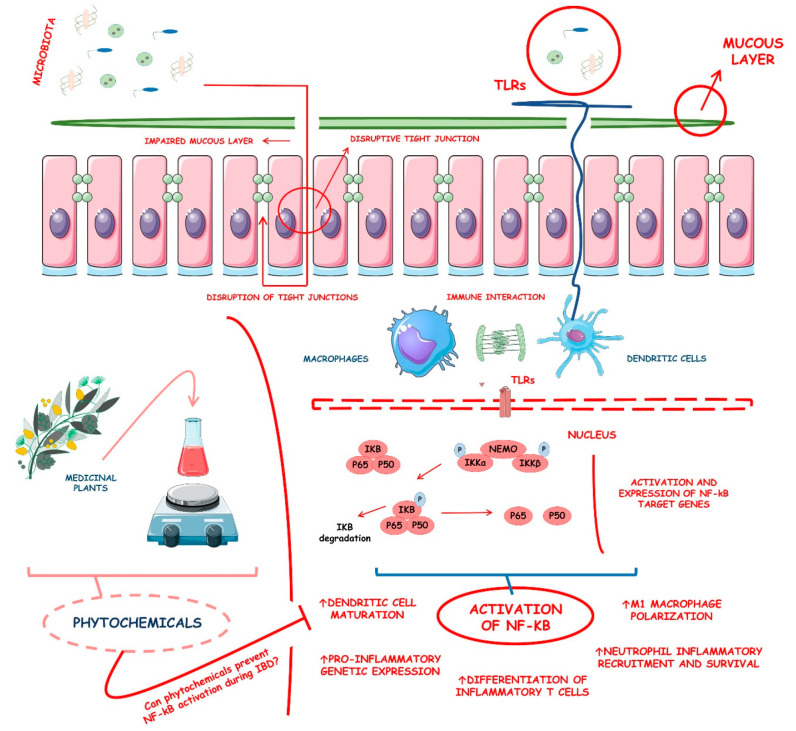
Inflammatory bowel diseases (IBD), activation of the nuclear factor-kB (NF-kB), and the possible roles of phytochemicals against this pathway activation during these diseases. ↑, increase; TLRs, Toll-like receptors; IKKα, IkappaB kinase alfa; IKKβ, IkappaB kinase beta; IKB, inhibitor of nuclear factor-kB.

IBD shows a fast-increasing incidence in many countries, and its prevalence is higher in Western regions, specifically Northern Europe and North America. In American adults, the prevalence reaches 1.3%, meaning that 3 million people are affected by this condition. These data make IBD be considered a global burden [5,6]. If it is not adequately treated, its course can severely damage the bowel leading to disability and a profound reduction in the quality of life and capacity to work. Affected patients can suffer from diarrhea, abdominal pain, bleeding, and many extra-intestinal manifestations. Therefore, the therapeutic approach targets the control of inflammation and OS, aiming for mucosal healing and reducing symptoms associated with a reduced risk of hospitalization [7,8]. Corticosteroids, aminosalicylates, antibiotics, immunosuppressive drugs, and monoclonal antibodies are usually used to treat patients. However, these drugs can be associated with several adverse effects, loss of efficacy, and high costs, and many patients are refractory to the treatment. For these reasons, many other substances have been considered to help treat IBD. Several phytochemicals, such as flavonoids, alkaloids, saponins, phenolic acids, and terpenoids, can be effective adjuvants for the therapeutic approach [9,10,11]. Figure 1 shows the main pathways involved in the pathophysiology of IBD and how the activation of the NF-kB pathways interferes with the occurrence of these diseases.

Phytochemicals can interfere with many targets, including NF-kB pathways [12,13,14,15]. Thus, they can be related to regulating many other processes and cascades of inflammatory responses. For these reasons, this review aims to investigate the effects of different phytochemicals in the NF-kB pathways in vitro and in vivo models of IBD. To the best of our knowledge, this is the first review to assess the roles of several phytochemicals in NF-kB regulation in IBD models.

## 2. Materials and Methods

### 2.1. Focal Question

The focal question that was considered to build this review was ‘’What are the Effects of Natural and Synthetized Phytochemicals as Regulators of the NF-kB Pathways in Inflammatory Bowel Diseases’’?

### 2.2. Language

Only studies originally written in English were included in this review.

### 2.3. Databases

PubMed and Google Scholar databases were searched. The mesh terms used were natural phytochemicals or synthesized phytochemicals or herbal medicine and ulcerative colitis or Crohn’s disease or inflammatory bowel diseases and nuclear factor kappa B. The mesh terms enabled the search and identification of in vivo and in vitro studies that were related to the objective of this review.

### 2.4. Study Selection

For this review, in vitro and in vivo studies were considered in the final analyses due to the lack of clinical studies regarding the use of phytochemicals as regulators of the NF-kB pathways in humans with IBD. Only full texts were considered.

### 2.5. Data Extraction

There was no restriction in the period for searching in vivo and in vitro studies. The included studies regarding using natural and synthesized phytochemicals as regulators of the NF-kB pathways in inflammatory disease models can be seen in Table 1.

### 2.6. Quality Assessment

The quality assessment of this narrative review was based on the initial literature review of the pathophysiology of UC and CD and activation of NF-kB, as well as its implications for IBD, and, furtherly, on the included in vivo and in vitro studies. The quality assessment was performed by two independent reviewers trained in the Scale for Assessment of Narrative Review Articles (SANRA), a scale of six different items proposed by Baethge et al. [16] that assesses the quality of non-systematic reviews.

**Table 1:** In vivo and in vitro studies evaluating phytochemicals’ roles as NF-kB regulators on inflammatory bowel diseases.

Phytochemicals	In Vivo/In Vitro Model(s)	Effective Dose(s)/ Concentration(s)	Related Clinical Symptoms of IBD	NF-kB-Related Dysregulation Indicators	Related Molecular Mechanisms in Regulation of NF-kB in IBD	**Ref.**
Curcumin 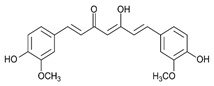	DSS-induced FVB/NJ mice model of colitis and NFκB-RE-Luc transgenic mice model of colitis	3 mg/day of TDNPs for 1 week orally	↑Inflammatory cells infiltration in the lamina propria, ↑epithelial erosion, ↑interstitial edema, and ↓colonic goblet cells	↑TNF-α, ↑IL-6, ↑IL-1β, ↑OS-related protein, ↓HO-1, and ↑MPO	↓NF-kB-p65-dependent luciferase activity, ↓phospho-NF-kB-p65 and ↓nuclear translocation of p65	[17]
	TNBS-induced Wistar Hannover rats model of colitis	20 mg/kg/day for 1 week orally	↑Inflammatory cells infiltration and ↑ulcer and granuloma formation	↑IL-6, ↑TNF-α, ↑MPO, ↑MDA, and ↑NO	↓NF-kB-related proteins expression and ↓oxidative-related enzymes expression	[18]
	DSS-induced BALB/c mice model of colitis	100 mg/kg mixed with olive oil in the chow	↓Body weight, ↑DAI, and ↓colon length	↑iNOS, ↑TNF-α, ↑IL-1β, ↑IL-6, ↑nitrite, and ↑S-nitrosylation of IKKβ	↓S-nitrosylation of IKKβ and ↓IκB phosphorylation	[19]
	TNBS-induced Sprague-Dawley rats model of colitis	100 mg/kg/day for 1 week orally	↑DAI score	↑MPO, ↑NF-kB mRNA, ↑IL-27, ↑TLR4 expression, ↑NF-kB-p65, and ↑IL-27 p28	↓NF-kB mRNA and ↓NF-kB-p65	[20]
	TNBS-induced Sprague-Dawley rats model of colitis	100 mg/kg and 200/mg/kg orally 2 h prior to induction of colitis	↓Body weight, ↑bloody diarrhea, thickened colon wall, ↓depletion of goblet cells, ↑hemorrhagic intestinal necrosis, ↑mucosal ulcerations, and ↑inflammatory cells infiltration	↑MPO and ↑NF-kB expression	↓NF-kB-related proteins expression and ↓oxidative-related enzymes expression	[21]
	Bacteria-induced Specific pathogen-free wild-type 129/SvEv mice and germ-free IL 10/mice models of colitis	0.1, 0.5, and 1% curcumin-supplemented diets for 5 days	↑Intestinal-associated ↑crypt hyperplasia, ↑lymphocytic and neutrophilic infiltrations, and ↑mucosal ulceration	↑IL-12/23p40, ↑IFN-γ, ↑NF-kB activation, and ↑pSer276-p65	↓IFN-γ and IL-12/23p40 genetic expression, ↓phospho-p65-positive expression, ↑IL-10 mRNA, ↓NF-kB-related proteins expression	[22]
	DNCB-induced Wistar rats model of colitis	25, 50, and 100 mg/kg/day of curcumin orally for 10 days	↑Intestinal ulcers, ↑inflammation in the colon, and ↓colon length	↑MPO, ↑LPO and ALP activities; ↑iNOS, and ↑NF-kB-related proteins expression	↓NF-kB-related proteins expression and ↓iNOS expression	[23]
	TNBS-induced Sprague–Dawley rats model of colitis	30 mg/kg/day intraperitoneally for 14 days	↑Intestinal epithelial necrosis, ↑glandular destruction, ↑inflammatory cells infiltration, ↓body weight	↑IL-1β mRNA, ↑TNF-α mRNA, ↑IFN-γ mRNA, ↑COX-2 mRNA, ↓PPAR-γ, ↓PGE_2_, ↑15d-PGJ_2_, ↓mRNA IL-4	↑mRNA do PPARγ, ↑15d-PGJ2, ↑PPAR-γ, ↓COX-2 mRNA, ↓IL-1β mRNA, ↓TNF-α mRNA, ↓IFN-γ mRNA, and ↑IL-4 mRNA	[24]
	TNBS-induced Wistar rats model of colitis	2% of curcumin mixed with the chow for 14 days	↓Body weight, intestinal ulcers, ↑inflammatory cells infiltration	↑NF-kB DNA ligation activity, ↑IkB degradation, ↑IL-1β and ↓IL-10	↓NF-kB DNA ligation activity, ↓IkB degradation, and ↓IL-1 β mRNA	[25]
	TNBS-induced BALB/c mice model of colitis	0.25% of curcumin mixed with the chow for 10 days	↓Body weight, ↑inflammatory cellular infiltration, and ↑mucosal and muscle damage	↑MPO, ↑IL-1 β, ↑NF-kB DNA ligation activity, and ↑p38 MAPK	↓NF-kB DNA ligation activity and ↓p38 MAPK	[26]
	TNBS-induced C3H mice model of colitis	25–300 mg/kg/day of curcumin orally for 10 days	↑Hemorrhagic and ulcerative damage to the distal colon, ↑mucosal congestion, ↑leucocyte cellular infiltrate in the submucosa, and ↓body weight	↑NO, ↑MPO, ↑MDA, ↑protease activities, ↑IFN-γ and IL-12 p40 mRNAs, and ↑iNOS	↓Serine protease activities, ↓IFN-γ and IL-12 p40 mRNA levels, ↓NF-kB-related proteins expression	[27]
	TNBS-induced C57BL/6 and BALB/c mice models of colitis	0.5%, 2.0%, or 5.0% of curcumin mixed with the chow for 7 days	↓Body weight, ↑crypts distortion, ↓goblet cells, and mononuclear cells infiltration	↑p65 nuclear expression, ↑IkB degradation, ↑macrophage infiltration, ↑IL-18, ↑NF-kB DNA ligation activity, ↑IL-6 mRNA, ↑IFN-γ mRNA, ↑TNF-α mRNA, and ↑IL-12 mRNA	↓IkB degradation, ↓NF-kB DNA ligation activity, ↓IL-6 mRNA, ↓IFN-γ mRNA, ↓TNF-α mRNA, and ↓IL-12 mRNA	[28]
Resveratrol 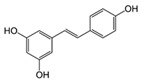	TNBS-induced C57BL/6 mice model of colitis	10 µL 4.5 mM and 10 µL 45 mM/day intraperitoneally for 4 days	Weight loss, diarrhea, bloody stool, ↑MPO activity, ↑DAI score, ↑colonic cytokine levels, and ↑visceral pain	↑pNF-kB, ↑TNF-α mRNA, ↑TNF-α, ↑TGF-β mRNA, ↑TGF-	↓pNF-Κb, ↓TNF-α mRNA, and ↓TGF-β mRNA	[29]
	LPS-treated Caco-2 cells	10–50 μM during 1 h of incubation	↑Colon inflammation measured by COX-2 and PGE_2_ expression levels	↑p65 nuclear translocation, ↑COX-2, ↑PGE_2_	↓p65 nuclear translocation, ↓IKK phosphorylation, ↓COX-2 mRNA, and ↓IkBα phosphorylation and degradation	[30]
	DSS-induced C57BL/6 mice model of colitis	100 µL of 10, 50, and 100 mg/kg on alternate days orally for 7 days	Colon inflammation (lymphocyte infiltration and distortion of glands), weight loss, and ↑serum pro-inflammatory cytokines	↑IL-6, ↑IL-1β, ↑IFN-γ, ↑TNF-α, ↑p-IkBα, ↓SIRT1	↓p-IkBα and ↑SIRT1	[31]
	DSS-induced ICR mice model of colitis	10 mg/kg/day orally for 7 days	↑Histopathological score	↑NF-kB-DNA binding complex, ↑IKKβ catalytic activity, ↑ERK phosphorylation, ↑iNOS expression, ↑STAT3	↓ERK phosphorylation, ↓NF-kB-DNA binding complex, and ↓IKKβ catalytic activity	[32]
	TNBS-induced Wistar mice model of colitis	10 mg/kg/day orally for 14 days	↑Macroscopic inflammation, presence of adhesions between the colon and small bowel and other organs, ulcers, crypt distortion, ↑leukocyte involvement, ↑pro-inflammatory cytokines production, and weight loss	↑MPO, ↑TNF-α, ↑NF-kB p65, ↑COX-2, ↑PGD_2_	↑Inflammatory mucosa cells apoptosis and ↓NF-kB p65	[33]
3-(4-Hydroxyphenyl)-propionic acid 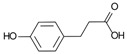	DSS-induced colitis in antibiotics-treated pseudo-germ-free mice and LPS-stimulated RAW264.7 cells	-	↑Intestinal inflammation and ↑OS both in vivo and in vitro	↑NF-kB-related activation proteins and ↑MAPK	↓NF-kB-related activation proteins and ↓MAPK	[34]
Sesamol 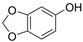	DSS-induced C57BL/6 mice model of colitis	100 mg/kg/day orally for 6 weeks	↑DAI, histopathological changes, and ↓intestinal barrier integrity	↑COX-2, ↑iNOS, ↑IL-6, ↑IL-1β, ↑TNF-α, ↑TLR4	↓COX-2 mRNA, ↓iNOS mRNA, ↓IL-6 mRNA, ↓IL-1β mRNA, ↓TNF-α mRNA, ↓TLR4 mRNA, and ↑p-NF-kB/NF-kB ratio	[35]
Kaempferol 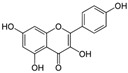	DSS-induced C57BL/6 mice model of colitis	50 mg/kg/day orally for 14 days	↑DAI, ↓colon length, ↑intestinal mucosal injury, and altered gut microbiota	↑IL-6, ↑IL-1β, ↑TNF-α, ↑IL-1β mRNA, ↑IL-6 mRNA, ↑TNF-a mRNA, ↑COX-2 mRNA, ↑MCP-1 mRNA, ↑iNOS mRNA, ↓IL-10 mRNA, ↑TLR4, ↑NLRP3, ↑MAPK1, ↑NF-kB-related proteins expression, ↓ZO-1, ↓occludin, and ↓claudin-1	IL-1β mRNA, ↓IL-6 mRNA, ↓TNF-a mRNA, ↓COX-2 mRNA, ↓MCP-1 mRNA, ↓iNOS mRNA, ↑IL-10 mRNA, ↓TLR4, ↓NLRP3, ↓MAPK1, ↓MyD88, ↓p-NF-kB-P65	[36]
Astragalin 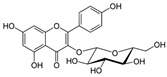	DSS-induced C57BL/6 mice model of colitis	200 µL of 50, 75, and 100 mg/kg/day orally for 7 days	↑DAI, ↑intestinal mucosal injury, ↑inflammatory cells infiltration, and ↓colon length	↑TLR4 mRNA,↑MCP-1 mRNA, ↑IL-1β mRNA, ↑TNF-α mRNA, ↑COX-2 mRNA, ↑IFN-γ mRNA, ↑p-IκBα, ↑p-IKKα/β, and ↑p-p65	↓TLR4 mRNA, ↑ZO-1 mRNA, ↑occludin mRNA, ↑Muc2 mRNA, ↓p-IκBα, ↓p-IKKα/β, and ↓p-p65, ↓MCP-1 mRNA, ↓IL-1β mRNA, ↓TNF-α mRNA, ↓COX-2 mRNA, ↓IFN-γ mRNA	[37]
	TNF-α -stimulated HCT-116 and HT-29 human colonic epithelial cells in vitro and DSS-induced C57BL/6 mice model of colitis in vivo	0, 20, 40, 60, 80, and 100 μM incubated for 24 h in vitro and 2 and 5 mg/kg/day orally for 7 days in vivo	↑Pro-inflammatory cytokines production and ↑colon cells proliferation in vitro and ↓colon length, ↑pro-inflammatory cytokines production, and weight loss in vivo	↑Cells proliferation, ↑TNF-α mRNA, ↑IL-8 mRNA, ↑IL-6 mRNA, ↑IκBα phosphorylation, and ↑NF-kB-DNA binding in vitro and ↑IκBα phosphorylation, ↑TNF-α mRNA, ↑IL-8 mRNA, and ↑IL-6 mRNA in vivo	↓Cells proliferation, ↓TNF-α mRNA, ↓IL-8 mRNA, ↓IL-6 mRNA, ↓IκBα phosphorylation, and ↓NF-kB-DNA binding in vitro and ↓IκBα phosphorylation, ↓TNF-α mRNA, ↓IL-8 mRNA, and ↓IL-6 mRNA in vivo	[38]
Pinocembrin 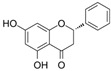	LPS-stimulated RAW264.7 and Caco-2 cells in vitro and DSS-induced C57BL/6 mice model of colitis in vivo	0–200 μM incubated for 24 h in vitro and 25, 50, and 100 mg/kg/day orally for 9 days in vivo	↑Inflammation in vitro and weight loss, ↑intestinal tissue damage, ↑mucosa muscle thickness, ↑neutrophil infiltration, ↑diarrhea, ↑microbiota alterations, and ↑blood in stool in vivo	↑TNF-α, ↑COX-2, ↑iNOS, ↑IFN-γ, ↑IL-6, ↑IL-15, ↑TLR4, ↑p65 phosphorylation, ↑IκBα phosphorylation, and ↓NO in vitro and ↑p65 phosphorylation, ↑TLR4 mRNA, ↑Myd88 mRNA, ↑iNOS mRNA, ↑COX-2 mRNA, ↑TNF-α mRNA	↓Pro-inflammatory cytokines expression, ↓NF-kB-luciferase activity and ↓TLR4/MD2 · LPS interaction in vitro and ↓TLR4 mRNA, ↓Myd88 mRNA, ↓iNOS mRNA, ↓COX-2 mRNA, ↓TNF-α mRNA, and ↓p65 phosphorylation in vivo	[39]
Oxyberberine 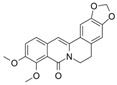 Bacterial metabolite	DSS-induced BALB/c mice model of colitis	12.5, 25, and 50 mg/kg/day orally/7 days	Shaggy hair, low vitality, body weight loss, diarrhea, occult fecal blood, and ↑DAI	↑MPO, ↓ZO-1, ↓ZO-2, ↓occludin, ↓JAM-A, ↓claudin-1, ↑IL-6, ↑IL-1β, ↑IL-17, ↑TNF-α, ↑IFN-γ, ↑TLR4, ↑MyD88, ↑p-IκBα, ↑p65 (nucleus), ↓IκBα, ↓p65 (cytoplasm)	↓MPO, ↑ZO-1, ↑ZO-2, ↑occludin, ↑JAM-A, and ↑claudin-1 expressions, ↓IL-6, ↓IL-1β, ↓IL-17, ↓TNF-α, and IFN-γ expressions, ↑p65 (cytoplasm), ↓p65 (nucleus), ↓p-IκBα/IκBα ratio, ↓TLR4, ↓MyD88, ↓NF-kB-p65 translocation, ↓IκBα phosphorylation	[40]
Berberine hydrochloride 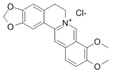	DSS-induced Wistar mice model of colitis	10, 30, and 50 mg/kg/day orally/6 weeks	Weight loss, ↓survival rate, ↓colon length, ↓colon weight, ↑DAI, ↓daily activity, anorexia, ↑inflammatory cells infiltration, ↑intestinal edema, and ↑microscopic damage scores	↑IL-1 mRNA, ↑IL-1β mRNA, ↑IL-6 mRNA, ↑IL-12 mRNA, ↑TNF-α, ↑IFN-γ mRNA, ↓IL-4 mRNA, ↓IL-10 mRNA, ↑iNOS, ↑MPO, ↑MDA, ↑p-NF-kB	↓IL-1 mRNA, ↓IL-1β mRNA, ↓IL-6 mRNA, ↓IL-12 mRNA, ↓TNF-α, ↓IFN-γ mRNA, ↑IL-4 mRNA, ↑IL-10 mRNA, ↓activity of iNOS, MPO, and MDA, ↓p-NF-kB, ↑p-STAT3 expression, ↑ZO-1 mRNA, ↑VCAM-1 mRNA, ↑occludin mRNA, and ↑claudin-1 mRNA	[41]
Berberine 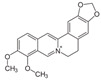	TNBS-induced C3H/HeN and C3H/HeJ mice models of colitis	10 and 20 mg dissolved in 2% Tween 80 solution/day orally/5 days	Intestinal inflammation measured by shortened, thickened, and erythematous colon	↑Lipid peroxidation, ↓SOD, ↓CAT, ↑TNF-α, ↑IL-1β, ↑IL-6, ↓IL-10, ↑iNOS, ↑COX-2, ↑TLR4, and ↑NF-kB activation (phosphorylation and nuclear translocation)	↓Lipid peroxidation, ↑antioxidant SOD, and CAT expressions, ↓pro-inflammatory cytokines TNF-α, IL-1β, and IL-6 expressions, ↑IL-10 expression, ↓iNOS, and ↓COX-2 activities, ↓TLR4 expression, and ↓NF-kB activation (phosphorylation and nuclear translocation)	[42]
Eriodictyol 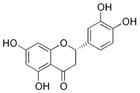	TNBS-induced Wistar mice models of colitis	5, 20, and 50 mg/kg/day orally/7 days	Weight loss, colon crypt destruction, mucosal ulceration, and colon inflammatory cells infiltration	↑MPO, ↑IL-6, ↑IL-1β, ↑IL 12, ↑IL-2, ↑TNF-α, ↓IL-10, ↓SOD, ↓CAT, ↓GSH-Px, ↑MDA, ↑TLR4, ↑p-IκBα, ↑p-p65, and ↓IκBα	↓MPO activity, ↓pro-inflammatory cytokines IL-6, IL-1β, IL-12, IL-2, and TNF-α expressions, ↑IL-10 expression, ↑antioxidant enzymes SOD, CAT, and GSH-Px expressions, ↓MDA expression, ↓p65 phosphorylation, ↓IκBα phosphorylation, and ↑IκBα	[43]
Betulin 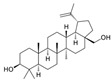	Acetic acid-induced Sprague Dawley mice models of colitis	8 mg/kg/day intraperitoneally for 14 days	Diffuse necrosis, congestion, and hemorrhage of the mucosal layer and submucosal edema, congestion, and immune/inflammatory cells infiltration	↑CRP, ↑LDH activity, ↑TLR4, ↑CD68 cells infiltration, ↑IL-6, ↑NF-kB expression, ↑TNF-α, ↑IL-1β, ↑caspase-3, and ↑caspase-8	↓LDH activity, ↓TLR4 content, ↓CD68 cells infiltration, ↓IL-6 content, ↓NF-kB expression, ↓TNF-α expression, ↓IL-1β, ↓caspase-3 expression, and ↓caspase-8 expression	[44]
Naringin 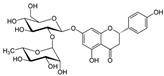	LPS-stimulated RAW264.7 cells in vitro and DSS-induced mice model of colitis in vivo	20 μM incubated for 1 h in vitro and 25, 50, and 100 mg/kg/day orally for 7 days in vivo	↑Intestinal inflammation in vitro and ↑intestinal mucosa injury and ↑DAI in vivo	↑TNF-α, ↑NF-kB activation, ↓PPARγ expression in vitro and ↑TNF-α, ↑IL-1β, ↑IL-6, ↑NF-kB-p65 phosphorylation, ↑IκB phosphorylation, ↓PPARγ expression, ↑MAPK, ↑NLRP3, ↑ASC, and ↑caspase-1 in vivo	↓TNF-α, ↓NF-kB activation, and ↑PPARγ expression in vitro and ↓pro-inflammatory cytokines TNF-α, IL-1β, and IL-6 expressions, ↓NF-kB-p65 phosphorylation, ↓IκB phosphorylation, ↑PPARγ expression, ↓phosphorylation levels of p38, ERK, and JNK, ↓NLRP3, ↓ASC, and ↓caspase-1 in vivo	[45]
5-Hydroxy-4-methoxycanthin-6-one 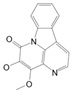	DSS-induced Sprague Dawley mice model of colitis	25, 50, and 100 mg/kg/day orally for 11 days	Weight loss, ↑DAI, ↓colon length, epithelial crypts destruction, disruption of the mucosal barrier, and massive submucosal infiltration of inflammatory cells	↑TNF-α, ↑IL-1β, ↑IL-6, ↓IL-10, ↓SOD, ↑MDA, ↑NF-kB/p65, ↑CD3, ↑MYD88, ↑p-IκBα, ↓IKKβ, ↓IκBα, ↑NF-kB/p65 nuclear translocation	↑SOD, ↓MDA, ↓TNF-α, ↓IL-1β, ↓IL-6 and ↑IL-10 expression levels, ↓NF-kB/p65 and ↓CD3 pro-inflammatory phenotypes, ↓NF-kB/p65 mRNA, ↓MYD88, ↓p-IκBα, ↑IKKβ and ↑IκBα proteins expression, ↓NF-kB/p65 nuclear translocation	[46]
Geniposide 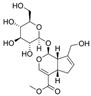	LPS-stimulated RAW264.7 cells in vitro and DSS-induced ICR mice model of colitis in vivo	200–1000 μM incubated for 24 h in vitro and 20 and 40 mg/kg/day orally/7 days in vivo	↓Cells viability in vitro and weight loss, ↑erosion and ↑distortion of crypts, ↑loss of glandular epithelium, and ↑inflammatory cell infiltration in vivo	↓SOD, ↑IL-1β, ↑IL-6, ↑TNF-α, ↑ROS, ↓HO-1, ↓Nrf2 activation, ↑p-NF-kBp65 and ↑p-IκBα in vitro and ↑MPO, ↓SOD, ↑IL-1β, ↑IL-6, ↑TNF-α, ↑inflammatory cells infiltration, ↓HO-1, ↓Nrf2 activation, ↑p-NF-kBp65 and ↑p-IκBα in vivo	↑SOD, ↓IL-1β, ↓IL-6, ↓TNF-α, ↓ROS, ↑HO-1, ↑Nrf2 activation, ↓p-NF-kBp65, and ↓p-IκBα in vitro and ↓MPO, ↑SOD, ↓IL-1β, ↓IL-6, ↓TNF-α, ↓inflammatory cells infiltration, ↑HO-1, ↑Nrf2 activation, ↓p-NF-kBp65 and ↓p-IκBα in vivo	[47]
Sesamin 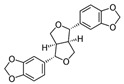	DSS-induced C57BL/6 mice model of colitis	50, 100, and 200 mg/kg/day orally/7 days	↓Colon length and ↓body weight	↑TNF-α, ↑IL-1β, ↑IL-6, ↑p-NF-kBp65, ↑p-IκBα, ↑NF-kB signaling and activity and ↑MAPK	↓TNF-α, ↓IL-1β, ↓IL-6, ↓p-NF-kBp65, and ↓p-IκBα expression levels, ↓NF-kB signaling and activity, and ↓MAPK levels	[48]
Taxifolin 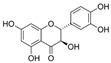	DSS-induced ICR mice model of colitis	100 mg/kg/day orally for 14 days	↑DAI, ↓colon length, ↓body weight, ↑crypt distortion, and ↑inflammatory cells infiltration	↑TNF-α, ↑IL-1β, ↑IL-6, ↓SIgA, ↓IL-10, ↓SOD, ↑p-NF-kB-p65 and ↑p-IkBα	↓TNF-α, ↓IL-1β and ↓IL-6 expression levels, ↑SIgA, ↑IL-10 and ↑SOD expression levels, ↓p-NF-kB-p65 and ↓p-IkBα	[49]
Isobavachalcone 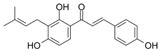	DSS-induced C57BL/6 mice model of colitis	25 and 50 mg/kg/day orally/4 days	↓Body weight, ↑DAI, ↑crypt distortion, ↑mucosal necrosis, ↑edema, ↑gland destruction, and ↑neutrophilic infiltration	↑MPO, ↑TNF-α, ↑IL-1β, ↑IL-6, ↑PGE2, ↑NO, ↑iNOS, ↑COX-2 and ↑p-NF-kB-p65	↓MPO, ↓TNF-α, ↓IL-1β, ↓IL-6, ↓PGE2, ↓NO, ↓iNOS and ↓COX-2 expression levels and ↓p-NF-kB-p65	[50]
d-pinitol 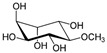	DSS-induced BALB/c mice model of colitis	10, 20, and 40 mg/kg/day orally/7 days	↓Body weight, ↑DAI, ↑ulcer formation, ↑thickened bowel wall, ↑hyperemia, ↑edema, and ↑mucosal inflammatory cells infiltration	↑MPO, ↑MDA, ↓GSH, ↓SOD, ↓CAT, ↑iNOS, ↑COX-2, ↑TNF-α, ↑IFN-γ, ↑IL-6, ↑IL-17, ↑IL-1β, ↓IL-10, ↓PPAR-γ and ↑NF-kB signaling	↓MPO, ↓MDA, ↑GSH, ↑SOD, ↑CAT, ↓iNOS, ↓COX-2, ↓TNF-α, ↓IFN-γ, ↓IL-6, ↓IL-17, ↓IL-1β, ↑IL-10, ↑PPAR-γ and ↓NF-kB signaling	[51]
Paeoniflorin-6’-O-benzene sulfonate 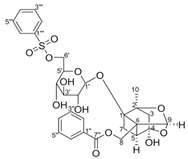	DSS-induced mice model of colitis	17.5, 35, and 70 mg/kg/day orally/6 days	↑M1 macrophage polarization and ↑intestinal barrier dysfunction	↑GRK2 activation and ↑TLR4-NF-kB-NLRP3 inflammasome signaling	↓GRK2 translocation and ↓TLR4-NF-kB-NLRP3 inflammasome signaling in macrophages	[52]
Thymol 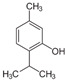	AcOH-induced Wistar mice model of colitis	10, 30, and 100 mg/kg/day orally/6 days	↑Intestinal inflammation and ↑OS	↑MPO, ↑TNF-α, and ↑p-NF-kB-p65	↓MPO, ↓TNF-α, and ↓p-NF-kB-p65	[53]
Tricin 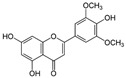	DSS-induced BALB/c mice model of colitis and LPS-induced RAW 264.7 treated cells	12.5, 25, and 50 µM incubated/30 min or 24 h in vitro and 100 and 150 mg/kg/day orally/7 days in vivo	↑ DAI, ↓Body weight, ↓colon length, ↑Inflammatory cells infiltration, ↑epithelial cell disorganization, ↑mucosal thickening, ↓crypts, ↑spleen weight, and ↑myeloid-derived suppressor cells (MDSC, CD11b^+^Gr1^+^), ↑MPO, ↑IL-6, TNF-α, and IL-1β in colonic tissues in vivo	↑NO, ↑IL-6, ↑TNF-α, ↑IL-1β, ↑MIP-2, ↑phosphorylated NF-kB-p65 in vitro	↓IL-6 expression, ↓TNF-α expression, ↓MIP-2 expression, ↓IL-1β expression ↓Phosphorylated nuclear p65 in vitro and ↓NF-kB pathway in vivo	[54]
Aesculin 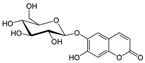	DSS-induced BALB/c mice model of colitis and LPS-induced RAW 264.7 treated cells	200, 300, 400, and 500 µM incubated for 1 h in vitro and 1 and 5 mg/kg/day intraperitoneally every two days after colitis induction for 12 days in vivo	↓Body weight, ↑DAI, ↑colon length, ↑colon weight, ↑inflammatory cells infiltration (mononuclear macrophages and neutrophils), ↑mucosal and submucosal lesion, ↑degeneration, and ↑crypt cells necrosis	↑p-p65, ↑IκBα phosphorylation and ↓PPAR-γ in vitro and ↑iNOS mRNA, ↑TNF-α mRNA, ↑IL-1β mRNA, ↑p-P65, ↑MAPKs protein and phosphorylation in vivo	↓TNF-α mRNA, ↓IL-1β mRNA, ↓p-P65, ↓IκBα phosphorylation, ↑PPAR-γ, ↓NK-kB activation in vitro and ↓iNOS mRNA, ↓TNF-α mRNA, ↓IL-1β mRNA, ↓NK-kB activation in vivo	[55]
Ginsenoside Rk3 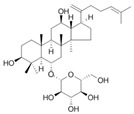	HFD-induced obese C57BL/6 mice model of colitis	30 and 60 mg/kg/day orally/8 weeks	↑Body weight, ↑fat accumulation, ↑glucose tolerance, ↓colon length, ↑inflammatory cells infiltration and ↑crypt lesions	↓ZO-1 mRNA, ↓claudin mRNA, ↓occludin mRNA,↑TLR4, ↑MYD88, and ↓IkBα	↑ZO-1 mRNA, ↑claudin mRNA, ↑occludin mRNA, ↓TNF-α mRNA, ↓IL-1β mRNA, ↓IL-6 mRNA, ↓MCP-1 mRNA, ↓F4/80 mRNA, ↓NADPH mRNA, ↓STAMP2 mRNA, ↓TLR4, ↓JNK/phosphorylation JNK, ↓NF-kB, ↓TLRA4/MYD88 pathway, and ↑IkBα mRNA	[56]
Lancemaside A 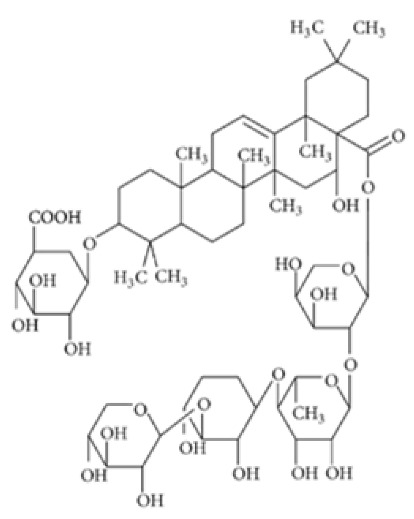	TNBS-induced ICR mice model of colitis and LPS-induced 293-hTLR4A-hemagglutinin treated cells	20 μM and 100μM incubated for 6 h in vitro and 10 or 20 mg/kg/day orally for 5 days in vivo	↓Colon length, ↑thicken, ↑erythematous colon, ↑edema, ↑inflammatory cells infiltration, and ↑epithelial ulcers	↑TLR4-linked NF-kB in vitro and ↑MPO, ↑TNF-α mRNA and ↑IL-1β mRNA, ↑IL-6 mRNA, ↑TLR4 mRNA, ↑NF-kB (pp65) mRNA, ↑COX-2 mRNA in vivo	↓TLR4-linked NF-kB in vitro and ↓TNF-α mRNA, ↓IL-1β mRNA, ↓IL-6 mRNA, ↓TLR4 mRNA, ↓NF-kB-p65 mRNA and ↓COX-2 mRNA in vivo	[57]
Tetramethylpyrazine 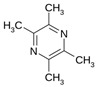	Oxazolone-induced KM mice model of colitis and LPS-treated Caco-2 cells	40 µg/mL incubated for 24 h in vitro and 80 mg/kg/day intraperitoneally/7 days in vivo	↓Body weight, ↑diarrhea with or without hematochezia, ↑DAI, ↑inflammation of the mucosa, ↑fibrotic thickening, ↑ulcers, ↑edema, ↑microhemorrhages, and ↑ necrosis	↑NF-kB translocation into the nucleus, ↓NF-kB P65 protein in the cytoplasm, ↑nuclear NF-kB P65 protein levels, ↑TNF- α, ↑IL-6, ↑IL-8, ↑INF-γ mRNA and ↑ROS in vitro, and ↑NF-kB P65 in the nucleus, ↓NF-kB in cytoplasmic, ↑C-MYC expression, ↑iNOS expression, ↑COX-2 expression in vivo	↓NF-kB translocation into the nucleus, ↑NF-kB P65 protein in the cytoplasm, ↓nuclear NF-kB P65 protein levels, ↓INF-γ expression, ↑P65 in the cytoplasm, ↓P65 in the nucleus in vitro and ↓NF-kB P65 in the nucleus, ↓p-IKBα, ↑NF-kB in the cytoplasm, ↓nuclear NF-kB p65 protein levels, ↓C-MYC expression, ↓iNOS expression and ↓COX-2 expression in vivo	[58]
Daurisoline 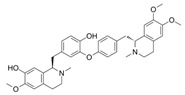	DSS-induced BALB/c mice model of colitis and LPS-induced RAW 264.7 treated cells	0, 0.5, 1, 2, 5, 10, 20, 50, and 100 μM incubated for 24 h in vitro and 10, 20, 40 mg/kg/day orally/7 days in vivo	↑DAI, ↑diarrhea, ↑bleeding, ↓colon length, ↑edema, ↑congestion, ↑thickening, ↑erosion, ↑ulceration, ↑adhesions to adjacent tissues, ↑mucosal damage, ↑inflammatory cell infiltration, ↑crypt loss, and ↑TUNEL stained spots	↑NO, ↑ROS, ↓GSH, ↑NF-kB-p65, ↑p65, ↓IkBα in vitro and ↑NO, ↑ COX-2, ↑PGE2, ↑IL-1β, ↑MMP-9, ↓IL-4, ↓IL-10, Gene expression of ↑Wnt-1, ↑β-Catenin, ↑cyclin-D1, ↑C-MYC, ↑Expression of Wnt-1, β-Catenin and LRP6, ↓Expression of p-GSK3β and ↑Expression of NF-kB p65 and p-IkBα in vivo	↓NF-kB p65, ↓p65, ↑IkBα in vitro and Gene expression of ↓Wnt-1, ↓β-Catenin, ↓cyclin-D1, ↓C-MYC, ↓GSK3β, ↑Expression of TCF-4, LEF-1 and p-GSK3β, ↓Expression of Wnt-1, β-Catenin and LRP6 and ↓expression of NF-kB p65 and p-IkBα in vivo	[59]
Tetrandrine 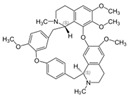	DSS-induced BALB/c mice model of colitis	40 mg/kg/day orally/7 or 14 days	↑DAI	↑NF-kB DNA binding activity, ↑IL-1β mRNA and protein, ↑TNF-α mRNA and protein, and ↑MPO	↓NF-kB DNA bindng activity, ↓IL-1β mRNA and protein, ↓TNF-α mRNA and protein	[60]
Diosgenin 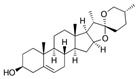	TNBS-induced Sprague-Dawley rat model of colitis	50, 100, or 200 mg/kg/day orally/14 days	↑DAI, ↓body weight, ↑colonic damage, ↑ulceration, ↑stool consistency score, ↑destruction of colon tissue, ↑inflammatory cell infiltration, ↑necrosis and ↑edema	↓GSH, ↓SOD, ↑MDA, ↑NO, ↑MPO, ↑hydroxyproline, ↑TNF-α, ↑IL-1β, ↑IL-6, ↓IL-10, ↑iNOs mRNA, ↑IFN-γ mRNA, ↑COX-2 mRNA, ↑LTB4 mRNA, ↑Bax, ↑Caspase-1, ↑NF-kB and ↑IκBα,	↓iNOs mRNA, ↓COX-2 mRNA, ↓IFN-γ mRNA, ↓Bax, ↓Caspase-1, ↓NF-kB and ↓IκBα	[61]
Mangiferin 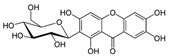	TNBS-induced C57BL/6 mice model of colitis and LPS-induced peritoneal macrophages	5, 10, and 20 μM incubated for 15 to 120 min in vitro and 10 or 20 mg/kg/day orally/3 days in vivo	↓Colon length, ↑MPO	↑IRAK1 phosphorylation and degradation, ↑degradation of IRAK1, 2, and 4, ↑NF-kB activation, ↑TAK1 phosphorylation and degradation, ↑IKKβ phosphorylation, ↑IκBα phosphorylation and degradation, ↑PGE_2_, ↑NO, ↑TNF-α expression, ↑IL-1β expression, ↑IL-6 expression, ↑IL-10 expression, ↑COX-2, ↑iNOS expression in vitro and ↑IRAK1 phosphorylation in vivo	↓IRAK1 phosphorylation and degradation, ↓NF-kB activation, ↓IKKβ phosphorylation, ↓IκBα phosphorylation and degradation, ↓p65 translocation, ↓MAPK p38 phosphorylation, ↓ERK phosphorylation, ↓JNK phosphorylation, ↓TNF-α expression, ↓IL-1β expression, ↓IL-6 expression, ↓COX-2 expression, ↓iNOS expression and ↑IL-10 expression in vitro and ↓phosphorylation of IRAK1 and IKKβ, ↓NF-kB activation, ↓TNF-α expression, ↓IL-1β expression and ↓IL-6 expression in vivo	[62]
Tryptanthrin 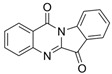	DSS-induced mice model of colitis	39.2, 78.4, and 156.8 mg/kg twice a day orally/8 days	↑CAS, ↓crypts and goblet cells, ↑erosive lesions, ↑inflammatory cell infiltration, and ↑atrophy	↑TNF-α, ↑IL-1β, ↑IL-6, ↓IL-10, ↑NF-kBp65, ↑p-STAT3, ↓IκBα protein, ↑STAT3 and ↑p-STAT3	↓NF-kBp65, ↓p-STAT3, and ↓IκBα degradation	[63]
l-Theanine 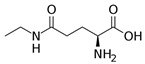	DSS-induced C57BL/6J mice model of colitis	Water contained 0.1% of l-theanine for 14 days orally	↓Body weight, ↓length of colon, ↓colon weight, ↑DAI, ↑inflammatory infiltrates, and ↑epithelial injury	↑TNF-α, ↑IL-1β, ↑IL-6, ↑COX2 mRNA, ↑iNOS mRNA, ↓Ki67-positive cells, ↑TUNEL-positive cells, ↓Occludin mRNA, ↓Claudin1 mRNA, ↓Ecadherin mRNA, ↑ p65, ↑p-p65, ↑p53, ↑p-p53 and ↑p-AKT expression and ↑lipid metabolic perturbation	↓COX2 mRNA, ↓iNOS mRNA, ↑Occludin mRNA, ↑Claudin1 mRNA, ↑Ecadherin mRNA, ↓p65, ↓p-p65, ↓p53, ↓p-p53, and ↓p-AKT expression	[64]
Koreanaside A	LPS-induced RAW 264.7 and peritoneal macrophages treated cells and DSS-induced ICR mice model of colitis	20, 40, or 80 µM in vitro incubated/4 days and 5 or 20 mg/kg/day orally/7 days in vivo	↑DAI, ↑body weight loss, ↑stool consistency, ↑occult fecal blood, ↓colon length, ↑spleen index, ↑mucosal layer, ↑ulceration, ↑crypt loss, and ↑inflammatory cell infiltration	↑NO, ↑PGE2, ↑expression of iNOS and ↑ expression of COX-2, ↑IL-6 mRNA, ↑TNF-α mRNA, ↑AP-1, ↑DNA-binding activity of NF-kB in vitro and ↑F4/80 mRNA, ↑Ly6G mRNA, ↓ZO-1 mRNA, ↓occludin mRNA, ↑claudin-1 mRNA, ↓E-cadherin mRNA, ↑N-cadherin mRNA, ↑vimentin mRNA, ↑iNOS mRNA, ↑COX-2 mRNA, ↑IL-6 mRNA, ↑TNF-α mRNA, ↑c-Fos, ↑p65, STAT1 and ↑STAT3 phosphorylation in vivo	↓iNOS expression and ↓COX-2 expression, ↓IL-6 mRNA, ↓TNF-α mRNA, ↓MyD88-dependent TLR4 pathway, ↓DNA binding of AP-1, ↓DNA-binding activity of NF-kB, ↓c-Fos phosphorylation, ↓phosphorylation and nuclear translocation of p65. ↓phosphorylation and degradation of IκBα, ↓phosphorylation of IKKα/β, ↓phosphorylation of TAK1, ↓STAT1 (Y701 and S727), ↓STAT3 (Y705), ↓JAK1 (Y1022), JAK2 (Y1007/1008) phosphorylation in vitro and ↓F4/80 mRNA, ↓Ly6G mRNA, ↑ZO-1 mRNA, ↑occludin mRNA, ↓claudin-1 mRNA, ↑E-cadherin mRNA, ↓N-cadherin, ↓iNOS mRNA, ↓COX-2 mRNA, ↓IL-6 mRNA, ↓TNF-α mRNA, ↓vimentin mRNA, ↓↑c-Fos, p65, STAT1 and ↑STAT3 phosphorylation in vivo	[65]
6-gingerol 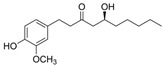	DSS-induced BALB/c mice model of colitis	100 and 250 mg/kg/day orally/14 days	↓Body weight, ↓crypt cells, ↓goblet, ↑granulation, ↑hyperplasia, and ↑inflammatory cells infiltration	↑IL-17, ↓IL-10, ↑IkBα, ↑p65, ↑p-IκBα and ↑p-p65	↓IkBα, ↓p65, ↓p-IκBα and ↓p-p65	[66]
Lycopene 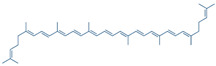	DSS-induced C57BL/6 mice model of colitis	5, 10, and 20 mg/kg/day orally/14 days	↓Body weight, ↑DAI, ↑colon length, ↑colon weight, ↑glandular disorder, and ↑inflammatory cell infiltration	↑MPO, ↓SOD, ↓ CAT, ↓GSH-Px, ↑MDA, ↑IFN-γ, ↑TNF-α, ↑IL-6, ↑IL-1β, ↑TLR4, ↑TRIF, and ↑p-NF-kB p65 expression, ↓ZO-1, ↓occludin, and ↓claudin-1 expressions	↓TLR4, ↓TRIF, ↓p-NF-kB p65 expression, ↑ZO-1, ↑occludin and ↑claudin-1 expressions	[67]
α-mangostin 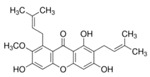	DSS-induced mice model of colitis	30 and 100 mg/kg/day orally/14 days	↓Body weight, ↑DAI, ↑diarrhea, ↑bleeding, ↓colon length, ↑ulceration, ↑erosion, ↑crypt distortion, ↑inflammatory cell infiltration, and ↑edema	↑MPO, ↑phosphorylation of IKKα and IκB, ↑activated NF-kB, ↑MAPK, ↑phosphorylation of ERK1/2, SAPK/JNK and p38	↓IKKα phosphorylation, ↓IκBα phosphorylation, ↓activated NF-kB, ↓phosphorylation of ERK1/2, SAPK/JNK and ↓p38	[68]
Ophiopogonin D 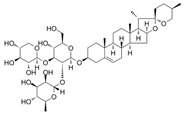	DSS-induced C57BL/6J mice model of colitis and LPS-induced IEC-6 treated cells	10 mg/kg and 40 mg/kg/day orally/7 days in vivo 20 μmol/L incubated for 24 h in vitro	↑Ulceration, ↑congestion, ↑edema, ↑inflammatory cell infiltration, ↓colon length, and ↓body weight	↑cl-caspase3 and ↑COX-2, ↑MLCK and ↑iNOS in vitro and ↑TNF-α, ↑IL-6, ↑IL-1β, ↓Bcl-2, ↓occludin, ↑NF-Κb-p65, ↑cl-caspase3, ↑Bax, ↑MLCK, ↑MDA, ↓GSH, ↓SOD, ↑iNOS, ↑COX-2 in vivo	↓NF-Κb-p65 in vivo and in vitro	[69]
Alantolactone 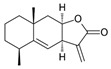	DSS-induced C57BL/6 mice model of colitis and LPS-induced RAW 264.7 and HT-29 colorectal treated cells	0–25 μM incubated for 2 h in vitro 50 mg/kg/day orally/9 days in vivo	↓Body weight, ↓bloody diarrhea, ↓colon length, ↑histological injury, ↑inflammatory cell infiltration	↑p-p65 nuclear translocation in vitro and ↑NF-kB p65 phosphorylation, ↑IκBα phosphorylation/degradation, ↑iNOS expression, ↑ICAM-1 expression, ↑MCP-1 expression, ↑COX-2 expression, ↑TNF-α expression, ↑IFN-γ expression, ↑IL-6 expression, ↑MPO, ↑ NO, ↑PGE2, ↑TNF-α, ↑IL-6 in vivo	↓p-p65 nuclear translocation, ↑hPXR via binding to hPXR-LBD in vitro and ↓NF-kB p65 phosphorylation, ↓IκBα phosphorylation/degradation, ↓iNOS expression, ↓ICAM-1 expression, ↓COX-2 expression, ↓TNF-α expression, ↓IFN-γ expression and ↓IL-6 expression in vivo	[70]
Sinomenine 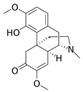	DSS-induced BALB/c mice model of colitis	30, 90, 270, 180, 540 mg/kg/day and 1.6 g/kg/day orally/9 days	↓Body weight, ↓food intake, ↑pasty stools, ↑DAI, ↓colon length, and ↑inflammatory cell infiltration	↑MyD88, ↑NF-kBp65, ↑TLR4, ↓SIGIRR expression, ↑TLR/NF-kB	↓MyD88 expression, ↓NF-kBp65 expression, ↓TLR expression, ↑SIGIRR expression, ↓TLR/NF-kB, ↓expression of IFN-γ, IL-1β, TNF-α, IL-6, and IL-12	[71]
Convallatoxin 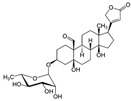	LPS-induced RAW264.7 and BMDMs macrophages and DSS-induced C57BL/6 mice model of colitis	10–50 nM incubated for 12 or 24 h in vitro and 50 or 150 μg/kg/day orally/10 days in vivo	↓Colon length, ↑colon and spleen weights, ↓body weight, ↑inflammatory cell infiltration, ↑ulceration, ↑necrosis, ↑congestion and ↑edema, ↑IL-1β, IL-6, and TNF-α in the colon	↑NF-kB, ↑COX-2, ↑iNOS, ↑ IL-1β, ↑ IL-6, ↑TNF-α, ↑NF-kB-p65 and ↓PPARγ in vitro and ↑COX-2, ↑iNOS, ↑IL-1β, ↑IL-6, ↑TNF-α, ↑nuclear NF-kB-p65, ↓PPARγ protein in vivo	↓NF-kB p65, ↑PPARγ, ↑PPARγ mRNA, ↓NF-kB mRNA, ↓IL-1β mRNA, ↓IL-6 mRNA, ↓TNF-α mRNA, ↓p-IκBα, ↑PPARγ siRNA in vitro and ↓nuclear NF-kB-p65, ↑PPARγ expression, ↓nuclear translocation of NF-kB-p65, ↓cytoplasmic p-IκBα expression, ↑PPARγ mRNA and ↓NF-kB mRNA in vivo	[72]
Fisetin 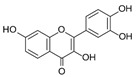	DSS-induced Balb/C mice model of colitis and LPS-induced macrophages treated cells	5 and 10 mg/kg/day orally/8 days in vivo and 0–50 μM incubated for 24 h in vitro	↑DAI, ↓body weight, ↓colon length, ↓crypts, ↓goblet cells, ↑inflammatory cell infiltration,	↑Nitrites, ↑TNF-α, ↑IL-1β, ↑IL-6, ↑COX-2, ↑iNOS, ↑NF-kB-p65 nuclear translocation, ↑IkBα phosphorylation and degradation in vitro and ↑MPO, ↑TNF-α, ↑IL-1β, ↑IL-6, ↑Nitrites, ↑COX-2, ↑iNOS, ↑nuclear NF-kB (p65), ↑phosphorylation of IκBα (p-IκBα/IκBα), ↑NF-kB (p65)-DNA binding activity, ↑p-p38/p38, ↑p-ERK/ERK, ↑Akt phosphorylation, ↓GSH, and ↑TBARS in vivo	↓NF-kB-p65e expression, ↓IkBα phosphorylation and degradation in vitro and ↓nuclear NF-kB (p65), ↓phosphorylation of IκBα (p-IκBα/IκBα), ↑NF-kB (p65)-DNA binding activity, ↓p-p38/p38, ↑p-ERK/ERK, and ↓Akt phosphorylation in vivo	[73]
Genipin 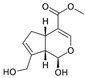	DSS-induced C57BL/6 mice model of colitis	2.5, 5, 10 mg/kg/day orally/14 days	↓Body weight, ↑intestinal epithelial destruction, ↑crypt abscesses, and ↑goblet cells loss	↑MPO, ↑MDA, ↑TNF-α, ↑IL-1β, ↑NF-kB signaling, ↓Nrf2 signaling and ↓HO-1	↓MPO, ↓MDA, ↓TNF-α and ↓IL-1β expression, ↓NF-kB signaling, ↑Nrf2 signaling and ↑HO-1 expression	[74]
Piperine 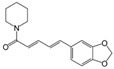	TNBS-induced Sprague–Dawley mice model of colitis	10, 20, and 40 mg/kg/day orally/14 days	↓Body weight, ↑colon weight-to-length ratio, and ↑ulceration	↑Oxide-nitrosative stress, ↑iNOS, ↑TNF-α, ↑IL-1β, ↑IFN-γ, ↑COX-2 mRNA, ↑LTB4, ↑IkBα, ↑NF-kB signaling, ↓occludin, ↓claudin-1, ↓zonula occludens-1, ↑caspase-1 and ↓IL-10	↓Oxide-nitrosative stress, ↓iNOS, ↓TNF-α, ↓IL-1β, ↓IFN-γ, ↓COX-2 mRNA, ↓LTB4 and ↓IkBα expression levels, ↓NF-kB signaling, ↑occludin, ↑claudin-1, ↑zonula occludens-1 and ↑IL-10 expression levels and ↓caspase-1	[75]
Ligustilide 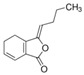	DSS-induced C57BL/6 mice model of colitis	15, 30, and 60 mg/kg/day orally/14 days	↓Body weight and ↓colon length, ↑diarrhea, ↑rectal bleeding, ↑ulceration, and ↑inflammatory cells infiltration	↑MPO, ↑iNOS, ↑TNF-α, ↑IL-1β, ↑IL-6, ↑IL-12, ↑MIP-1α, ↑IL-17, ↓PPARγ and ↑NF-kB-p65	↓MPO, ↓iNOS, ↓TNF-α, ↓IL-1β, ↓IL-6, ↓IL-12, ↓MIP-1α and ↓IL-17 expression levels, ↑PPARγ expression and signaling and ↓NF-kB-p65 expression	[76]
Evodiamine 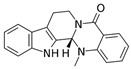	DSS-induced C57BL/6 mice model of colitis	20, 40, and 80 mg/kg/day orally/10 days	↑Diarrhea, ↑fecal bleeding, ↑colon shortening, and ↓body weight	↑MPO, ↑TNF-α, ↑IL-1β, ↑IL-6, ↑p-NF-kB p65, ↑p-IkB, ↑NLRP3, ↑ASC, ↑caspase-1, ↓ZO-1 and ↓occludin	↓MPO, ↓TNF-α, ↓IL-1β, ↓IL-6, ↓p-NF-kB p65, ↓p-IkB, ↓NLRP3, ↓ASC, ↓caspase-1, ↑ZO-1 and ↑occludin	[77]
Chrysin 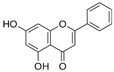	TNBS-induced C57BL/6 mice model of colitis	25 mg/kg/day orally/10 days	↓Body weight, ↑diarrhea, ↑fecal bleeding, ↑crypt distortion, and ↑inflammatory exudate	↑p-65, ↑IkBα phosphorylation and degradation, ↑NF-kB nuclear translocation, ↑iNOS mRNA, ↑ICAM-1 mRNA, ↑MCP-1 mRNA, ↑COX-2 mRNA, ↑TNF-α mRNA, ↑IL-6 mRNA, and ↑MPO	↓p-65, ↓IkBα phosphorylation and degradation, ↓NF-kB nuclear translocation, ↓iNOS mRNA, ↓ICAM-1 mRNA, ↓MCP-1 mRNA, ↓COX-2 mRNA, ↓TNF-α mRNA, ↓IL-6 mRNA, and ↓MPO	[78]
Wogonoside 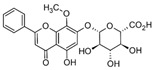	DSS-induced BALB/c mice model of colitis and LPS-induced Human acute monocytic leukemia THP-1 treated cells	12.5, 25, or 50 mg/kg/day orally/10 days in vivo and 0.1 mM incubated for 4 h in vitro	↓Body weight, ↓colon length, ↓spleen weight, ↑inflammatory cell infiltration, ↑ulcers, ↑edema and ↑congestion, ↑CD11b+ F4/80+ macrophages and ↑CD11b+ Gr-1+ neutrophils,	↑NLRP3 mRNA and ↑pro-caspase-1 mRNA in vitro and ↑IL-1β, ↑TNF-α, ↑IL-6, ↑NF-kB p65, ↑cleaved caspase-1 (p10), ↑cleaved-IL-1β, ↑NLRP3 and ↑ASC in vivo	↓IL-1β mRNA, ↓TNF-α mRNA, ↓IL-6 mRNA, ↓NF-kB nuclear translocation, ↓IkBa phosphorylation, ↓phosphorylation of p65, ↓NF-kB DNA binding activity, ↓NLRP3 mRNA and ↓pro-caspase-1 mRNA in vitro and ↓NF-kB, ↓NF-kB-p65, ↓IkBa phosphorylation, ↓p65, ↓p65 phosphorylation and ↓NF-kB DNA binding activity in vivo	[79]
Oxymatrine 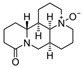	TNBS-induced rats model of colitis	10, 30, or 60 mg/kg/day intraperitoneally/7 days	↓Body weight, ↓colon length, ↑DAI, ↑ulcers, ↓goblet cells, and ↑inflammatory cell infiltration	↓ZO-1 mRNA, ↓occludin mRNA, ↓claudin-2 mRNA, ↑IL-6, ↑TLR9, ↑Myd88 and ↑p-NF-kB P65	↓IL-1β mRNA, ↓TNF-α mRNA, ↓IL-6 mRNA, ↓NF-kB, ↓TLR9 expression, ↓Myd88, ↓TLR9/Myd88/NF-kB pathway	[80]
Epicatechin 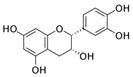	DSS-induced C57BL/6J mice model of colitis and LPS-induced RAW264.7 and IEC-6 treated cells	100, 200, or 300 mg/kg/day orally/7 days in vivo and 0.1 µM, 1 µM or 10 µM incubated for 4 h in vitro	↓Body weight, ↓colon length, ↑intestinal bleeding, ↑DAI, and ↑CMDI scores	↑TNF-α, ↑IL-6, ↑NO, ↑MPO and ↑NF-kB	↓NF-kB expression	[81]
Thymoquinone 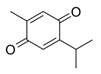	DSS-induced C57BL/6J mice model of colitis and TNF-α-induced HT-29 treated cells	20 and 40 mg/kg/day orally for 8 days in vivo and 0, 12.5, 50, 100, 150, and 200 µM incubated for 24 h in vitro	↑DAI, ↑inflammatory cells infiltration, ↑MPO, ↓crypts, ↓villi, ↑submucosal edema, and ↑epithelium destruction	↑CXCL-1 mRNA, ↑IL-8 mRNA and COX-2 mRNA in vitro and ↑IL-1β expression, ↑TNF-α expression, ↑IL-6, expression ↑IL-6 mRNA, ↑IL-1β mRNA, ↑TNF-α mRNA, ↑COX-2, ↑iNOS, ↑COX-2 mRNA, ↑iNOS mRNA, ↑p-ERK, ↑p-JNK, ↑p-p38, ↑phosphorylation of the NF-kB protein and ↓PPAR-γ expression in vivo	↓CXCL-1 mRNA, IL-8 mRNA, and COX-2 mRNA, ↑PPAR-γ expression both at protein and mRNA in vitro and ↓IL-6 mRNA, ↓IL-1β mRNA, ↓TNF-α mRNA, ↓p-ERK, ↓p-JNK, ↓p-p38, ↓phospho-NF-kB protein and ↑PPAR-γ in vivo	[82]
Fraxinellone 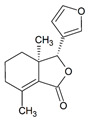	DSS-induced C57BL/6J mice model of colitis and LPS-induced Human THP-1 treated cells	7.5, 15, 30 mg/kg/ day intraperitoneally/9 days in vivo and 10, 30 µM incubated for 24 h in vitro	↓Body weight, ↑diarrhea, ↑loose feces, ↑visible fecal blood, ↑mortality, ↑gross bleeding, ↑ulcerations, colon length, ↑DAI, ↑inflammatory cell infiltration at mucosa and submucosa, ↑crypts distortion, and ↓goblet cells	↑IL-1β, ↑IL-18, ↑TNF-α, ↑IL-6 in vivo and ↑IL-1β, ↑IL-18 and ↑NO in vitro	↓VCAM1 mRNA, ↓iNOS mRNA, and ↓COX-2 mRNA in vivo and ↓IL-1β expression, ↓IL-18 expression, ↓phosphorylation of IKKα/β, ↓IκBα, ↓phosphorylation of the p65, ↓p65, ↓Caspase-1 activation and ↓NLRP3 inflammasome in vitro	[83]
Artesunate 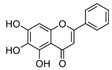	DSS-induced Sprague-Dawley rats model of colitis and LPS-induced RAW264.7 treated cells	10, 30, and 50 mg/kg/day orally/5 days in vivo and 5, 10, and 20 µg/mL incubated for 24 h in vitro	↑DAI, ↓hemoglobin, ↓colon length, and ↑cell destruction	↑TNF- α, ↑IL-8, ↑IFN-γ, ↑TLR4, ↑p-NF-kB, ↑p-p38, ↑Bax, ↑caspase-9 and ↓Bcl-2	↓TLR4, ↓p-NF-kB, ↓p-p38, ↓Bax, ↓caspase-9 and ↑Bcl-2	[84]
Aesculetin 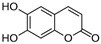	DSS-induced C57BL/6J mice model of colitis and LPS-induced RAW264.7 treated cells	20 mg/kg/day orally/7 days in vivo and 10, 25, 50 µM incubated for 4 h in vitro	↓Colon length, ↓body weight, ↑DAI, and ↑inflammatory cell infiltration	↑NO, ↑iNOS expression, ↑p–NF–κB-P65 expression, ↑NF-kB P65 nuclear translocation, ↑p38 phosphorylation, ↑JNK phosphorylation, ↑ERK phosphorylation, ↑NLRP3 expression in vitro and ↑NF-kB P65, ↑TNF-α and ↑IL-6 in vivo	↓iNOS expression, ↓p–NF–κB P65 expression, ↓NF-kB-P65 nuclear translocation, ↓p38 phosphorylation, ↓JNK phosphorylation, ↓ERK phosphorylation and ↓NLRP3 expression in vitro and ↓NF-kB-P65, ↓p38 phosphorylation, ↓JNK phosphorylation and ↓ERK phosphorylation in vivo	[85]
Euphol 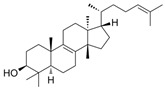	DSS and TNBS-induced CD1 mice model of colitis and LPS-induced BMDMs treated cells	3, 10, and 30 mg/kg twice a day orally for 3, 4 or 7 days in vivo and 1 and 10 µM incubated for 24 h in vitro	↑Hemorrhage in the colonic lumen, ↓body weight, ↑diarrhea with bloody stools, ↑DAI, ↑mucosal neutrophils infiltration, ↓crypts, ↓goblet cells, ↑mucosal hyperemia, ↑mucosal necrosis	↑IL-1β, ↑CXCL1, ↑MIP-2, ↑MCP-1, ↑IL-1β mRNA, ↑CXCL1 mRNA, ↑TNF-α mRNA, ↑IL-6 mRNA, ↑NOS2 expression, ↑VEGF expression, ↑Ki67 expression, ↑NF-kB-p65 phosphorylation, ↑ICAM-1 mRNA, ↑VCAM-1 mRNA and ↑LFA-1 mRNA	↓NOS2 expression, ↓VEGF expression and ↓p65 NF-kB activation	[86]
Nobiletin 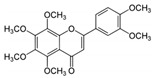	TNBS-induced Sprague-Dawley rats model of colitis and LPS-induced Caco-2 treated cells	20 and 40 mg/kg/day orally/7 days in vivo and 0, 10, 20, 40, or 80 incubated for 0–36 h µM in vitro	↑DAI, ↓body weight, ↑colon weight-to-length Ratio, ↑intestinal permeability, ↑MPO, ↑TNF-α, ↑IL-1β, ↑IL-6, ↑NO, ↑PGE2, ↑iNOS expression, ↑COX-2 expression in vivo	↑Akt, ↑MLCK mRNA, ↑MLCK protein and ↑NF-kB p65 protein expression in vitro and ↑MLCK, ↑NF-kB, ↑PI3K, ↑Akt and ↑NF-kB p65 protein Expression in vivo	↓Akt, ↓MLCK mRNA, ↓MLCK protein and ↓NF-kB p65 protein expression in vitro and ↓MLCK, ↓NF-kB, ↓phosphatidylinositol 3-kinase (PI3K), ↓Akt, ↓NF-kB p65 protein expression, ↓iNOS expression and ↓COX-2 expression in vivo	[87]
Galangin 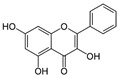	DSS-induced Swiss albino mice model of colitis	40 mg/kg/day orally for 20 days	↑Mucosal ulceration, ↑mucosal necrosis, ↑inflammatory cell infiltration in the lamina propria and submucosa	↑TLR4 mRNA, ↑NF-kB p65 nuclear translocation, ↑TNF-α and ↑IL-6	↓TLR4 mRNA, ↓NF-kB-p65 nuclear translocation, ↓TNF-α expression, and ↓IL-6 expression	[88]

↑, increase; ↓, decrease; AcOH, acetic acid; ALP, alkaline phosphatase; Akt, protein kinase b; AP-1, activating protein-1; ASC, apoptosis-associated speck-like protein; Bax, Bcl-2-associated X protein; Bcl-2, B-cell lymphoma 2 protein; BMDMs, bone-marrow-derived macrophages; Caco-2, human colorectal adenocarcinoma cells; CAS, Clinical activity score; CAT, catalase; CD1b, CD1b T cell surface glycoprotein; CD11b, integrin alpha M; CD3, cluster of differentiation 3, c-Fos, cellular proto-oncogene Fos; cl-caspase3, cleaved Caspase-3; CMDI, colon macroscopic damage index; C-MYC, cellular myelocytomatosis oncogene; COX-2, cyclooxygenase 2; CRP, c reactive protein; CXCL-1, chemokine (C-X-C motif) ligand 1; DAI, disease activity index; DMSO, dimethylsulfoxide; DNA, deoxyribonucleic acid; DNCB, dinitrochlorobenzene; DSS, dextran sulfate sodium; ERK, extracellular signal-regulated kinase; GRK2, G-protein-coupled receptor kinase 2; GSK3β, glycogen synthase kinase 3 beta; GSH, glutathione; GSH-Px, glutathione peroxidase; HCT-116, human colorectal carcinoma cell line; HFD, high-fat diet; HO-1, heme oxygenase-1; hPXR, human pregnane X receptor; HT-29, human colorectal adenocarcinoma cell line; ICAM-1, Intercellular Adhesion Molecule 1; ICR, Institute of Cancer Research; IEC-6, intestinal epithelioid cell 6; IFN-γ, interferon gama; IκBα, nuclear factor of kappa light polypeptide gene enhancer in B-cells inhibitor alpha; IKK, multi-subunit IkB kinase; IKKβ, inhibitor of nuclear factor kappa-B kinase subunit beta; IL-1, interleukin 1; IL-1β, interleukin 1 beta; IL-2, interleukin 2; IL-4, interleukin 5; IL-6, interleukin 6; IL-10, interleukin 10; IL-12, interleukin 12; IL-15, interleukin 15; IL-17, interleukin 17; IL-18, interleukin 18; IL-27, interleukin 27; iNOS. inducible nitric oxide synthase; IRAK1, interleukin 1 receptor associated kinase 1; JAM-A, junctional adhesion molecule A; JAK1, Janus kinase 1; JAK2, Janus kinase 2; JNK, c-Jun N-terminal kinases; Ki67, antigen KI-67; KM, Kunming Mouse; LEF-1, lymphoid enhancer binding factor 1; LFA-1, lymphocyte function-associated antigen 1; LDH, lactate dehydrogenase; LPO, lactoperoxidase; LPS, lipopolysaccharide; LRP6, low-density lipoprotein receptor-related protein 6; LTB4, leukotriene B4; Ly6G, lymphocyte antigen 6 complex locus G; MAPK, mitogen-activated protein kinase; MAPK1, mitogen-activated protein kinase 1; MCP-1, monocyte chemoattractant protein-1; MDA, malonaldehyde; MD2, myeloid differentiation protein 2; MDSC, myeloid-derived suppressor cells; MIP-1α, macrophage inflammatory protein 1 α; MIP-2, macrophage inflammatory protein-2; MMP-9, matrix metalloproteinase-9; MLCK, myosin light-chain kinase; MPO, myeloperoxidase; mRNA, messenger RNA; Muc2, Mucin 2, oligomeric mucus/gel-forming; MyD88, MYD88 innate immune signal transduction adaptor; NADPH, nicotinamide adenine dinucleotide phosphate; NF-kB, nuclear factor kappa b; NF-kBp65, NF-kB classical signaling pathway protein; NLRP3, NLR [nucleotide-binding domain leucine-rich repeat] family pyrin domain containing 3; NO, nitric oxide; NOS2, nitric oxide synthase 2 (inducible); Nrf2, nuclear factor erythroid 2–related factor 2; OS, oxidative stress; p-, phosphorylation; p-AKT, phosphorylated protein kinase B; PGD_2_/PGE_2_, prostaglandin D2; PGE_2_, prostaglandin E2; PGJ2, prostaglandin J2; PI3K, phosphoinositide 3-kinase; pNF-kB, phospho-NF-kB p65; PPARγ, peroxisome proliferator- activated receptor gamma; ROS, reactive oxygen species; RSV, resveratrol; SAPK, stress-activated protein kinases; STAMP2, six transmembrane protein of prostate 2; STAT1, signal Transducer And Activator Of Transcription 1; SIRT1, NAD-dependent deacetylase sirtuin-1; SIgA, immunoglobulin A; SIGIRR, single Ig IL-1-related receptor; SOD, superoxide dismutase; STAT3, signal transducer and activator of transcription-3; TAK1, transforming growth factor-β-activated kinase 1; TBARS, thiobarbituric acid reactive substances; TCF-4, transcription factor 4; TDNPs, turmeric-derived nanoparticles; TGF-β, transforming growth factor beta; THP-1, human leukemia monocytic cell line; TLR4, toll-like receptor 4; TLR4-NF-kB-NLRP3, toll-like receptor 4-nuclear factor kappa b-NLR family pyrin domain containing 3; TLR9, toll-like receptor 9; TNBS, 2,4,6-trinitrobenzene sulfonic acid; TNF-α, tumor factor necrosis alfa; TRIF, TIR-domain-containing adapter-inducing interferon-β; VCAM-1, vascular cell adhesion protein 1; VEGF, vascular endothelial growth factor; Wnt-1, proto-oncogene Wnt-1; ZO-1, zonula occludens-1; ZO-2, zonula occludens-2.

## 3. Results

This manuscript comprised data from 56 different phytocompounds and their derived secondary metabolites on in vitro and in vivo models of IBD. One study was only in vitro, 46 were in vivo, and 25 were both in vitro and in vivo studies. The included in vitro studies used many different cell models of IBD (such as RPMI 1640 treated Colon-26 cells, DMEM-treated Caco 2BBE, RAW 264.7 treated cells, LPS-treated human colorectal adenocarcinoma cells (Caco-2) cells, TNF-α-stimulated human colorectal carcinoma cell line (HCT-116) and human colorectal adenocarcinoma cell line (HT-29), and 2,4,6-trinitrobenzene sulfonic acid (TNBS) -induced C3H/HeN). In addition, the in vivo models were a dextran sulfate sodium (DSS)-induced mice model of colitis, NFκB-RE-Luc transgenic models of colitis, TNBS-induced mice model of colitis, TNBS-induced Wistar Hannover rats model of colitis, TNBS-induced Wistar Hannover rats model of colitis, and Acetic acid-induced mice models of colitis. The included phytochemicals were curcumin, resveratrol, 3-(4-hydroxyphenyl)-propionic acid, sesamol, kaempferol, astragalin, pinocembrin, oxyberberine (bacterial metabolite), berberine hydrochloride, berberine, eriodictyol, betulin, naringin, 5-hydroxy-4-methoxycanthin-6-one, geniposide, sesamin, taxifolin, isobavachalcone, d-pinitol, paeoniflorin-6′-O-benzene sulfonate, thymol, tricin, aesculin, ginsenoside Rk3, lancemaside A, tetramethylpyrazine, daurisoline, tetrandrine, diosgenin, mangiferin, tryptanthrin, l-theanine, koreanaside A, 6-gingerol, lycopene, α-mangostin, ophiopogonin D, alantolactone, sinomenine, convallatoxin, fisetin, genipin, piperine, ligustilide, evodiamine, chrysin, wogonoside, oxymatrine, epicatechin, thymoquinone, fraxinellone, artesunate, aesculetin, euphol, nobiletin, and galangin.

## 4. Discussion

### 4.1. Physiopathology of Ulcerative Colitis

Pathologically, UC involves the intestinal epithelial barrier, gut commensal microbiota, and the immune system. The disruption of the tight junctions and the mucus film covering the lumen intestinal epithelial barrier layer promotes a state of increased permeability of the intestinal epithelium to luminal agents. At this point, commensal bacteria start to invade the other layers of the bowel’s tissues, and macrophages and dendritic cells, cells of the innate immune system, begin to recognize the bacterial antigen of the commensals through the utilization of pattern recognition receptors, such as the called Toll-like receptors (TLRs). Thus, these cells get over a tolerogenic status to an active phenotype in which activating pro-inflammatory pathways stimulates the transcription of multiple pro-inflammatory genes to promote the production and secretion of many pro-inflammatory cytokines like TNF-α, IL-12, IL-23, IL-1Β, and IL-6 [89,90,91].

After the initial processing of the commensal bacterial antigens, the stimulated dendritic cells and macrophages start to present the antigens to naïve cluster of differentiation (CD) 4 (CD4) T cells, which are the main ones responsible for promoting T cells differentiation into Th (T helper) 2 effector cells, which are characterized by the production of a specific interleukin, which is the IL-4. Although naïve CD4 T cells are the primary regulators of the immune response, many other immune cells are involved pathogenically in UC development. For example, the natural killer (NK) cells are the main source of IL-13 during the disease process. This interleukin has been associated with the massive disruption of the intestinal lumen epithelial cell barrier. The inflamed intestine starts to express more and more adhesive molecules to leukocytes, leading to the increased entry of T cells into the intestinal wall lamina propria. Moreover, the intestinal cells have up-regulation of pro-inflammatory chemokines like C-X-C motif chemokine ligand (CXCL) 1 (CXCL1), CXCL3, and CXCL8, which only perpetuates the inflammatory cycle of colitis [89,90,91,92].

### 4.2. Physiopathology of Crohn’s Disease

CD is a chronic inflammatory intestinal condition that was first considered regional ileitis, with increasing incidence worldwide. However, the evolution of the molecular and histopathological assays showed that CD inflammation could affect the whole intestine, although the most frequently affected part is the distal ileum. CD patients experience periods of flares and remissions during their disease course. Besides other pathogenic events, the inflammatory burden of inflammatory cells maintaining the active disease is well known as the most important event during CD pathogenesis. For these reasons, the treatment aims are to stop the inflammation cascade by eliminating the production of pro-inflammatory cytokines in the bowel [93,94,95,96].

As innate immunity is involved in the defects in the intestinal mucous barrier, adaptative immunity relies on a Th1 lymphocytic immunological response mediated by the production of pro-inflammatory cytokines, such as TNF-α, IL-12, IL-23, and IL-34. Treg cells also mediate the production of pro-inflammatory cytokines during CD development. The inflammatory response is sustained by the migration of mainly of these Th1 lymphocytes and Treg cells to the sites of affection by the interaction with integrins (like the receptor for the α4β4 integrin) and other adhesion molecules (like the leucocyte MAcCAM-1) mediated by the presence of multiple different chemokines and metalloproteinases (such as the matrix metalloproteinase (MMP) 1 (MMP-1) and MMP-3, produced by stimulation of leukocytes’ proteins like CD44 and CD26) [93,95,97].

### 4.3. NF-kB and Its Related Molecular Insights into Inflammation

NF-kB, or nuclear factor of the κ-chain in B-cells, is an inducible transcription factor that regulates many different genes involved in developing and regulating inflammatory and immunomodulated processes. NF-kB is composed of five structurally-related members, which are the NF-kB1 (p50), NF-kB2 (p52), ReIA (p65), ReIB, and c-ReI. These entities mediate the transcription of mainly inflammatory factors by binding to specific deoxyribonucleic acid (DNA) elements named kB enhancers and forming various hetero- and homo-dimers. NF-kB-related proteins are sequestered in the induced cells’ cytoplasm by a family of inhibitory proteins called IkB, characterized by the presence of ankyrin repeats. The most studied member is the nuclear factor of kappa light polypeptide gene enhancer in B-cells inhibitor alpha (IkBα). Generally, the NF-kB’s vital activators are pro-inflammatory cytokines (IL-1β, TNF-α, IL-12, IL-33, IL-17, granulocyte macrophage-colony stimulating factor (GM-CSF), and lymphotoxin-β), bacterial antigens (lipopolysaccharides (LPS) such as flagellin, CpG-DNA, and enterotoxins), viral agents (viral proteins), many receptor ligands (CD40L, B-cell activating factor (BAFF), Fas ligand (FasL), hepatocyte growth factor (HGF), bone morphogenetic proteins (BMP) 2 (BMP-2), BMP-4, and TNF-related apoptosis inducing ligand (TRAIL)), cell lysis products (damage-associated molecular patterns (DAMPs), high mobility group box 1 (HMGB1), extracellular RNA molecules, and extracellular DNA molecules), eukaryotic parasites (*Candida albicans* and *Leishmania*), physiological stresses (endoplasmic reticulum (ER) stress, OS, acidic pH, hyperglycemia), physical stress (ionizing radiation and ultraviolet (UV)-light), and presence of modified proteins and particles (advanced glycation end products (AGEs), oxidized low-density lipoprotein cholesterol (LDL), and amyloid protein fragments) [98,99,100].

Molecularly, NF-kB activation involves two major signaling pathways, the canonical and the noncanonical (or alternative). Canonically, the NF-kB pathways are activated through several stimuli such as ligands of various pro-inflammatory cytokines receptors, T cell receptor (TCR), B cell receptor (BCR), TNF receptor (TNFR) superfamily member, and pattern-recognition receptor (PRRs). To enter the canonical pathway, NF-kB starts with the inducible degradation of IkBα, which is triggered by its site-specific phosphorylation by a multi-subunit IkB kinase (IKK) complex. The IKK complex can be activated by pro-inflammatory cytokines, growth factors, microbial components, mitogens, and stress agents and is formed of two catalytic subunits, which are the IKKα and IKKβ, additionally to a regulatory subunit called NF-kB essential modulator (NEMO) or IKKγ. Upon activation, the IKK complex phosphorylates IKKα in two N-terminal serines. At this point, IKK triggers the ubiquitin-dependent IKKα in a proteasome that translocates rapidly and transiently the canonical NF-kB members from the cell’s nucleus. These canonical members are the p50/ReIA and the p50/c-ReI dimers [98,101,102,103].

In turn, the non-canonical (alternative) NF-kB activation is more selective. This pathway responds only to a specific stimuli group, including the ligands of a subset of the TNFR superfamily members (Lymphotoxin beta receptor [LTβR], tumor necrosis factor receptor superfamily member 13C (BAFFR), receptor activator of nuclear factor kappa-Β (RANK), and CD40). Different from the canonical, the non-canonical pathway does not involve the degradation of the IkBα molecules but relies on processing the NF-kB2 (p100) precursor, an NF-kB-related protein. The processing of the p100 consists of the degradation of its C-terminal IkB-like structure, which results in the generation of a mature NF-kB2 p52 and the nuclear translocation of the NF-kB complex p52/ReIB. This degradation process is mediated by a central signaling molecule called NF-kB-inducing kinase (NIK), which functionally activates the IKKα and cooperates with its mediation on p100 phosphorylation. At this point, the phosphorylation of the p100 induces the p100 ubiquitination and further processing into the aforementioned NF-kB2 p52 and its nuclear translocation [98,104,105,106].

Theoretically, the canonical activation of the NF-kB is involved mainly in the development and progression of immunological responses. On the other hand, the non-canonical NF-kB activation is more involved in the supplementation signaling axis that cooperates with the canonical activation of the pathway in regulating specific adaptative functions of the adaptive immune system. To target inflammation, NF-kB not only exerts increases in the production of pro-inflammatory cytokines, adhesion molecules, and chemokines but also regulates the immune cells’ differentiation, apoptosis, morphogenesis, and proliferation. Dendritic cells are stimulated to maturation. T cells are stimulated to inflammatory-mediated differentiation and activation and to form memory T cells principally through the actions of IL-12, IL-23, and RORγt, which are factors directly related to the NF-kB signaling. Macrophages are stimulated to massive production of pro-inflammatory cytokines and chemokines, in addition, to polarizing into M1. Neutrophils are stimulated into anti-apoptosis states and massive recruitment into inflammation sites [98,101,105,107].

In other words, NF-kB induces inflammatory cells survival due to the production of anti-apoptotic factors (such as Fas, B-cell leukemia/lymphoma 2 protein (Bcl-2), caspases, BFL-1, survivin, and cellular FLICE (FADD-like IL-1β-converting enzyme)-inhibitory protein (c-FLIP)), cell cycle regulators (like Bcl-2-like protein 1 (Bcl-2L1), Plasminogen activator inhibitor-2 (PAI2), and cyclin) and inflammatory cells proliferation due to the production of cells cycle regulators (IL-1, TNF-α, IL-2, IL-8, and IL-12). NF-kB also augments inflammatory cells’ adhesion and invasion into inflammatory sites due to augmentation of adhesion molecules production, such as intercellular adhesion molecule 1 (ICAM-1), epithelial cell adhesion molecule-1 (ECAM-1), vascular cell adhesion molecule 1 (VCAM-1), MMPs, and selectin. NF-kB-related pro-inflammatory cytokines and chemokines (monocyte chemoattractant protein-1 (MCP-1), IL-18, CXCL1, CXCL10, macrophage inflammatory proteins (MIP) 2 (MIP-2), and regulated upon activation, normal T cell expressed and secreted (RANTES)) also work on inducing angiogenesis in areas near inflammatory sites that help modulate the inflammatory response [98,101,104,105].

Biologically, NF-kB has a major function corresponding to changing cellular programs across different types of stress and harmful situations. During NF-kB activation and signaling, different cells are stimulated to cope with the threat and activate their defense mechanisms to profoundly eliminate the endangering and escape death with the final aim of returning to the original physiological state in which all cells of the body are organized into homeostasis. That is why NF-kB targets to upregulate or induce a variety of genes to elucidate its responses [101]. Due to its inflammatory nature and role in the body’s defense, NF-kB is implicated in the physiopathology and progression of several inflammatory diseases, such as IBD [98,108,109].

NF-kB triggers inflammatory diseases mainly by contributing to the activation and regulation of different inflammasomes. As a group of intracellular multi-protein complexes, inflammasomes assemble activating caspases principally in response to the presence of DAMPs and pathogen-associated molecular patterns (PAMPs). Inflammasomes are integrative parts of innate immunity and regulate the gut microbiota composition (a primary reason why NF-kB is involved with IBD). They are composed of a ligand-sensing receptor (usually a member of the nucleotide-binding domain leucine-rich repeat (NLR) family NLRP1, NLRP3, NLRC4, or the absent in melanoma 2 [AIM2]), one adaptor protein (potentially the apoptosis-associated speck-like protein containing CARD), and a pro-caspase (generally, the pro-caspase 1). Upon appropriate stimulation, the inflammasome receptors start to oligomerize and recruit pro-caspases 1 via apoptosis-associated Speck-like protein (ASC). At this point, pro-caspases 1 are converted into active caspases 1, and cleave the pro-IL-1β and pro-IL-18 into their mature forms. Then IL-1β and IL-18 are secreted as pro-inflammatory cytokines, and most inflammasome-derived inflammatory processes begin. The NLRP3 is the most extensive inflammasome ever studied, and it is composed of the NLRP3, ASC, and never in mitosis gene A (NIMA)-related kinase 7 (NEK7, an essential regulatory protein), and pro-caspases 1. When dysbalanced and disrupted, NF-kB super-activates inflammasomes, and an inflammatory disease arises [98,108,110].

### 4.4. NF-kB and Its Implications on IBD

NF-kB is implicated in the pathogenesis of IBD insofar as this transcription factor turned out to be the primary regulatory component of the inflammatory burden during the intestinal inflammation during UC and CD. The NF-kB signaling is not usually activated in regular patients but is activated in the inflamed bowel of patients affected by IBD. Immunofluorescence staining assays demonstrated that NF-kB activation in the intestinal lumen is predominant in the intestinal macrophages and epithelial cells of the intestinal lumen mucosa and, interestingly, the higher the number of cells with activated NF-kB stain, the worse the severity of the intestinal inflammation. In addition to macrophages and epithelial cells, other parts of the intestinal mucosa can express the NF-kB signaling pathways, as in the lamina propria fibroblasts, which are also assumed to play pro-inflammatory stimuli during IBD [111,112].

Especially in intestinal macrophages, NF-kB expression is accompanied by increased production of pro-inflammatory cytokines, such as TNF-α, IL-1, and IL-6, due to the actions of NF-kB in expanding the capacity of these cells to produce inflammatory mediators. In other intestinal cells, NF-kB also regulates the expression of IL-12 and IL-23. Predominantly, the NF-kB-derived pro-inflammatory cytokines are responsible for two mechanisms involved in the development of IBD lesions. Firstly, the produced pro-inflammatory cytokines directly mediate mucosal tissue damage by principally up-regulation of matrix metalloproteinases production and secretion in the intestinal lumen. Secondly, NF-kB-derived pro-inflammatory cytokines also mediate the stimulation, activation, and differentiation of immunological cells derived from lamina propria of the bowel mucosa, resulting in chronic inflammation and, therefore, in the perpetuation of mucosal damage. In this case, NF-kB acts crucially during principally CD development, in which Th1 cells are stimulated due to the major production of TNF-α and IL-23, and the TNF-α potentializes the proper NF-kB in a kind of positive feedback [111].

Besides the roles of NF-kB in macrophage-derived inflammatory patterns, which are well established, the actions of the signaling pathway in epithelial cells during the setting of IBD are still controversial. IL-6 and TNF-α majorly activate the epithelial cells at the beginning of the inflammatory process, leading to IBD. NF-kB is then activated, and this activation can be demonstrated by the increased intestinal lumen epithelial expression of a different cluster of adhesion molecules, such as the ICAM-1, which helps in the leukocyte recruitment to the inflammatory intestinal sites, such as in the case of neutrophils granulocytes. NF-kB also affects colonic lamina propria fibroblasts via activation of T-cell-derived CD40 ligand (CD40L). The CD40L interacts with the CD40 receptor expressed by the colonic fibroblasts on their surface and, thereby, induces the activation of the pro-inflammatory NF-kB in these cells, stimulating the increase in the expression of many different pro-inflammatory cytokines, such as IL-6, IL-8, and chemoattractant molecules, such as the monocyte chemoattractant protein [111].

Mainly in UC, recent research has revealed that immunological and inflammatory mechanisms are not unique in exerting the biological structural changes of colonic intestinal epithelial cells during the ulcerative phases of the disease. Ferroptosis is a condition of the iron-dependent type of nonapoptotic cell death that has recently emerged as a regulatory mechanism of the necrosis process observed in UC patients. Iron accumulation plays a critical role in cell death due to the increased accumulation of Reactive Oxygen Species (ROS) and the derived lipid peroxidation. Morphologically, ferroptosis appears as shrunken mitochondria, condensed mitochondrial membranes, and reductions in the mitochondrial crista. Additionally, ferroptosis also is related to a downregulation of the antioxidant systems, such as glutathione peroxidase (GPX) 4 (GPX4), which corresponds to a potent scavenger of reactive lipid molecules, and of the up-regulation of prostaglandin-endoperoxide synthase 2 (PTGS2). Many authors suggested that a specific subunit of the NF-kB, the Bp65 subunit (NF-kBp65), plays a role in ferroptosis inhibition insofar as the deletion of intestinal epithelial cells NF-kBp65 was an up-regulator of ferroptosis that can exacerbate intestinal inflammation. There are suggestions that phosphorylated NF-kBp65 significantly inhibits endoplasmic reticulum stress signaling in the intestinal cells directly by binding a factor called eukaryotic initiation factor 2α. In this case, the ferroptosis process can be inhibited or tremendously decelerated [113].

Considering that upregulated NF-kB signaling pathway plays a critical role in IBD progression, Han et al. [114] hypothesized that different NF-kB activation levels influence CD’s clinical manifestations during the patient’s disease course. The results showed that higher NF-kB activation showed higher frequencies of ileocolonic involvement and lower frequencies of perianal lesions compared to patients with low NF-kB activation. Furthermore, higher activation demonstrated an association with higher histological scores. Lower NF-kB activity did not significantly affect disease progression course or outcome, principally after surgical treatment procedures.

Another important form of cell death that compromises the mucosal barrier integrity during IBD is pyroptosis; a caspase-1-dependent programmed cell death. This phenomenon features the gasdermin D (GSDMD) cleavage and causes the translocation and plasma membrane rupture of the mucosal cells, resulting in the massive release of pro-inflammatory cytokines to activate immunological mediators like IL-1β and IL-18. During IBD development, principally IL-1β works to alter the intestinal epithelial tight junctions, which results in increased intestinal permeability. Moreover, IL-18 has been shown to contribute to the decrease in the gut mucosal barrier integrity, causing inflammation and amplifying tissue damage. Mechanistically, pyroptosis can depend on a highly glycosylated transmembrane protein called CD147 to happen. CD147 induces pyroptosis in intestinal epithelial cells by enhancing the expression of IL-1β and IL-18, which activate inflammasomes, including caspase-1 and GSDMD, aggravating inflammatory reactions and events. It is known that CD147 stimulates the phosphorylation of NF-kBp65 in intestinal epithelial cells. Thus, inhibition of the CD147 molecule can correspond to a novel therapeutic strategy to combat IBD [112,115].

There is a long list of studies regarding the inhibition of NF-kB as a powerful treatment strategy for IBD. Qiu et al. [116] found that maresin 1, a specialized resolving mediator of inflammation derived from macrophages, has a powerful anti-NF-kB inhibition effect through toll-like receptor 4 (TLR4), which alleviated UC in a rat model of dextran sulfate sodium-induce disease. Wang et al. [117] found by studying a rat model of acetic acid-induced UC that a methane-rich saline preparation could effectively promote an anti-inflammatory response against the disease by principally mediating the blockage of the NF-kB through TLR4 again. Tong et al. [118] found that milk alleviates colitis by regulating Treg cells and inhibiting TLR4/ NF-kB signaling pathways, restoring immunity balance and reshaping gut microbiota. Chen et al. [119] found that sodium butyrate inhibits inflammation in a Trinitrobenzenesulfonic Acid (TNBS)-induced IBD mice model and maintains epithelium barrier integrity through activation of G protein coupled receptor 109A (GPR109A) and inhibition of the protein kinase B (Akt) and NF-kBp65 signaling pathways.

### 4.5. Phytochemicals That Influence the NF-kB Signaling during IBD: An Overview

As shown in the following items of this section, many phytochemicals have a critical role in regulating inflammatory processes observed in IBD. Table 1 summarizes some of these molecules and their actions

#### 4.5.1. Curcumin

*Curcuma longa* (CL) is a plant belonging to *Zingiberaceae*, also known as turmeric. Widely used as a spice in food preparation, mainly in India, CL is grown in Southeast Asia, China, and Latin America. Polyphenolic bioactive compounds, called curcuminoids (CCMs), such as curcumin (CUR), demethoxycurcumin (DMC), and bisdemethoxycurcumin (BMC), were identified from this plant. Curcumin has therapeutic potential for anti-inflammatory, anti-diabetic, anti-cancer, and anti-aging effects [120]. In addition, studies have revealed that this compound can act therapeutically against chronic pulmonary, cardiovascular, neurological, neoplastic, psychological, and metabolic diseases [121]. CUR plays anti-inflammatory and immunomodulatory roles in the pathogenesis and progression of IBD. Studies showed that it stimulates the differentiation of intestinal Treg cells, and in experimental mouse models, these Tregs prevented the development of colitis. Other research indicated that pretreatment with CUR suppressed LPS-induced NF-kB-p65 translocation and mitogen-activated protein kinases (MAPK) phosphorylation in dendritic cells of colitis mice models, reducing inflammation [122].

Liu et al. [17] evaluated the effects of turmeric-derived nanoparticles containing high levels of curcumin in DSS-induced FVB/NJ female mice and NFκB-RE-Luc transgenic mice model of colitis, in addition to RPMI 1640-treated Colon-26 cells, DMEM-treated Caco-2BBE and RAW 264.7 cells. The results showed that NF-kB translocation was inhibited in vitro and in vivo. In addition, the treatment decreased Myeloperoxidase (MPO) levels and mRNA expression of pro-inflammatory cytokines such as TNF-α, IL-1β, and IL-6.

Altinel et al. [18] aimed to investigate the roles of anal and oral CUR against NF-kB activity using a mouse model of TNBS-induced colitis. Compared to the control group, colonic levels of NF-kB had a more significant reduction with oral CUR. Furthermore, the expression of platelet-derived growth factor (PDGF) and TNF-α also lower in the oral CUR group when analyzed by biochemistry.

An in vivo study by Kao et al. [19] analyzed the effects of CUR on NF-kB deactivation in a murine model of DSS-induced colitis. The results showed that CUR could inhibit IKKβ activity by S-nitrosylation in colonic cells, a nitric oxide (NO) dependent modifier at the cysteine residue that regulates inducible nitric oxide synthase (iNOS) activity. In addition, CUR was able to sequentially inhibit the phosphorylation of IkB, actions that culminated in the inhibition of NF-kB in the intestine. CUR also repressed the production of pro-inflammatory cytokines such as TNF-α, IL-1β, and IL-6.

An in vivo study performed by Zeng et al. [20] with a murine model of TNBS-induced colitis evaluated the protective effects of CUR against the production of inflammatory factors in the intestine of the animals. The CUR-treated group showed a reduction in NF-kB and IL-27 mRNA expression compared to the untreated group. In addition, there was a significant reduction in NF-kB-p65 protein expression in the group that used CUR.

According to Lubbad et al. [21] study, using CUR in an in vivo model of TNBS-induced colitis resulted in decreased colonic activation of NF-kB. In addition, CUR decreased colonic concentrations of oxidative enzymes such as Malondialdehyde (MDA) and MPO.

Larmonier et al. [22] indicated that there was no concentration of CUR that modified NF-kB activation in the subepithelial tissue of IL-10^−/−^/NF-kBEGFP transgenic mice. However, 0.1% CUR demonstrated protective effects against colitis and decreased NF-kB activation in colonic epithelial cells only in IL-10^−/−^ mice.

Venkataranganna et al. [23] used NCB-02, a CL extract with high concentrations of CUR, to assess its benefits on dinitrochlorobenzene (DNCB) -induced UC in rats. Results showed that NCB-02 inhibited the expression of NF-kB, iNOS, and MPO in treated UC mice. There were also improvements in colon weight and size and reduced damage to the gut structure.

Zhang et al. [24] induced TNBS colitis in an in vivo model to assess the effects of CUR on NF-kB deactivation. The animals treated with CUR had much smaller and more superficial ulcers than the untreated group. Furthermore, most of these ulcers were in an advanced state of regeneration. The group treated with CUR still showed a decrease in intestinal epithelial necrosis and infiltration of inflammatory cells in the submucosa and lamina propria of the intestine. Peroxisome proliferator-activated receptor γ (PPARγ) messenger RNA (mRNA) was increased in the colonic mucosa in the CUR-treated group, and CUR also increased the concentration of Prostaglandin E2 (PGE2) and 15-deoxy-delta12,14-prostaglandin J2 (15d-PGJ2). Finally, there were reductions in mRNA expression of IL-1β, TNF-α, and IFN-γ, cyclooxygenase-2 (COX-2).

The experimental study by Jian et al. [25] in a rat model of TNBS-induced colitis evaluated the DNA binding activity of NF-kB. Rats were treated with CUR, stimulating IkB degradation and inhibiting IL-1β mRNA expression.

Ukil et al. [27] evaluated the effects of CUR on TNBS-induced colitis in mice and showed that sick animals presented an increase in NF-kB DNA binding activity in nuclear extracts from inflamed colonic tissue. CUR inhibited the binding of the NF-kB factor to its nuclear targets. Such CUR action was due to the inhibition of NF-kB mobility. It was also observed that the pre-treatment group with CUR showed a greater formation of Th2 response by suppressing IFN-y and IL-12 p40 mRNA and reducing the expression of iNOS mRNA. Serine protease activity, as well as neutrophilic infiltration and accumulation of MPO, MDA, and NO were also prevented by pretreatment with CUR.

Using a model of DNB-induced colitis in mice, Salh et al. [26] demonstrated that CUR leads to a reduction in NF-kB DNA binding activity and reduces MPO action and neutrophilic infiltration, as well as IL-1β mRNA expression.

Sugimoto et al. [28] evaluated the effects of CUR on TNBS-induced colonic inflammation in mice and showed a decrease in p65 expression and inhibited IkB degradation and NF-kB translocation in the nucleus of intestinal epithelial cells. Furthermore, CUR inhibited IkB degradation in colonic macrophages and reduced colonic inflammation by decreasing the infiltration of CD4 T cells into the lamina propria and the expression of pro-inflammatory cytokine genes in the colonic mucosa.

**Figure 2:**
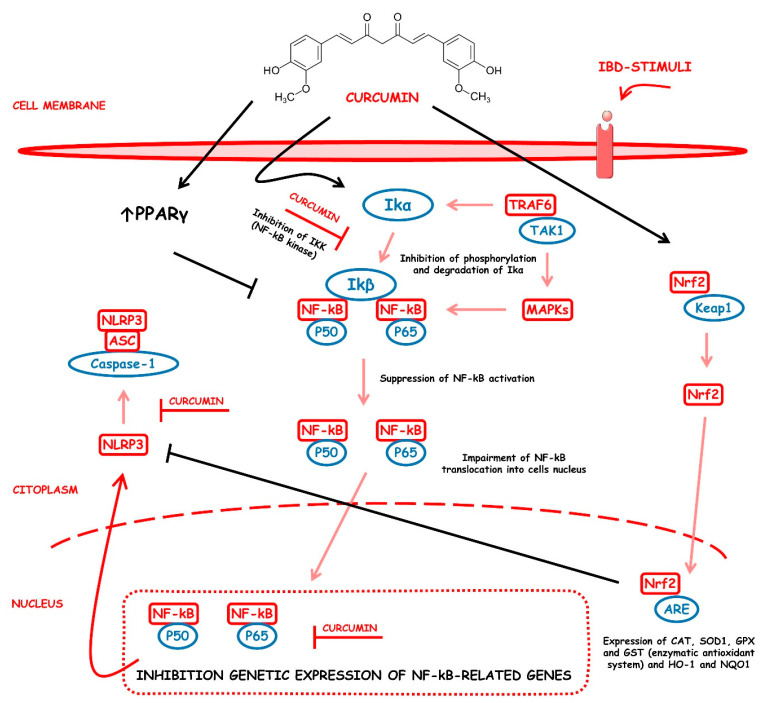
NF-kB regulation with the use of curcumin. ↑, increase; ARE, antioxidant response elements; ASC, apoptosis-associated Speck-like protein; CAT, catalase; HO-1, heme oxygenase 1; GPX, glutathione; GST, glutathione S-transferases; IBD, inflammatory bowel diseases; Keap1, Kelch-like ECH-associated protein 1; MAPK, mitogen-activated protein kinases; NF-kB, nuclear factor-Kb; NLRP3, NLR (/nucleotide-binding leucine-rich repeat receptor) family pyrin domain containing 3; NQO1, NAD(P)H dehydrogenase (quinone) 1; Nrf2, nuclear factor erythroid 2–related factor 2; SOD1, superoxide dismutase 1; TAK1, transforming growth factor-β-activated kinase 1; TRAF6, tumor necrosis factor receptor (TNFR)-associated factor 6.

Figure 2 shows the regulation of NF-kB pathways with the use of curcumin.

#### 4.5.2. Resveratrol

Resveratrol (RSV) is a phenolic compound mainly produced by plants in response to environmental stress. Primarily, RSV is considered an anti-inflammatory phytochemical insofar as it inhibits many pro-inflammatory pathways, including the NF-kB, and exerts antioxidant, anti-aging, and cardiovascular protection effects. This phenol has been encountered in more than 72 different plant species until now and exists in two geometric isomers, which are the trans- and cis-, and two glucosides, which are the trans- and cis- piceids [123,124].

In a mice model of TNBS-induced colitis, Lu et al. [29] showed that RSV supplementation leads to NF-kB downregulation through decreases in pNF-Κb, TNF-α mRNA, TNF-α, transforming growth factor beta (TGF-β) mRNA, and TGF-β. RSV also induced visceral hyperalgesia, an effect derived from the inhibition of spinal NF-kB signaling, which reduced the production of pro-inflammatory cytokines.

Cianciulli et al. [30] demonstrated the down-regulation of NF-kB through the decreases of p65 nuclear translocation, COX-2 mRNA expression, COX-2 and PGE_2_, IKK phosphorylation, and IkBα phosphorylation and degradation in vitro study conducted with LPS-treated Caco-2 cells as a model of colitis.

Singh et al. [31] showed that RSV significantly inhibited NF-kB through decreasing p- (phosphorilated) IkBα and increasing NAD-dependent deacetylase sirtuin-1 (SIRT1) genetic expression. SIRT1 inversely correlates with SIRT1 expression during IBD treatment against DSS-induced colitis in mice.

Youn et al. [32] demonstrated that RSV treatment significantly inhibited the activation of the NF-kB signaling via decreases in extracellular signal-regulated kinase (ERK) phosphorylation, NF-kB-DNA binding complex, and IKKβ catalytic activity.

Martín et al. [33] evaluated the role of RSV treatment against TNBS-induced colitis in a mice model. The results showed significant inhibition of the NF-kB via decreases in TNF-α, NF-kB p65, and COX-2 expression, as well as up-regulation of inflammatory mucosa cell apoptosis.

**Figure 3:**
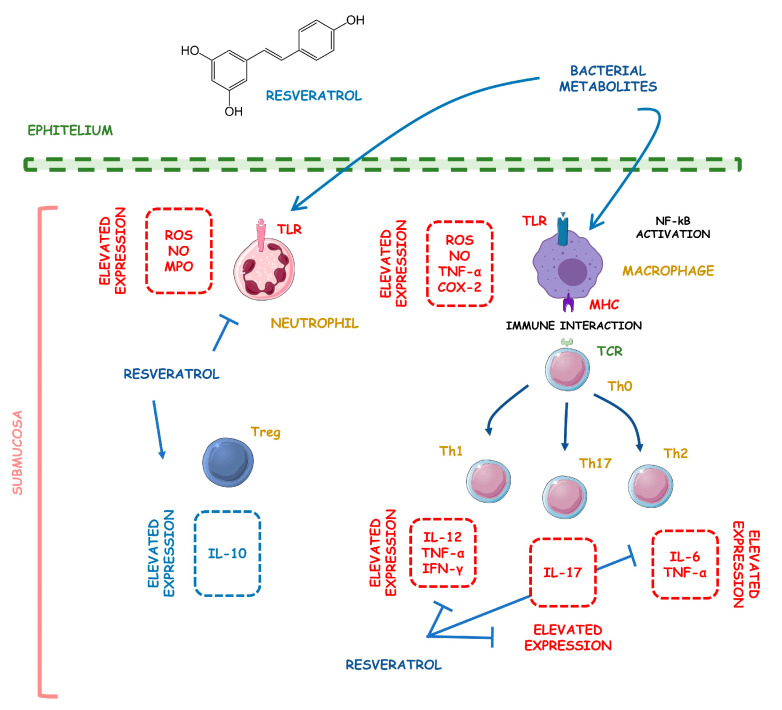
Main regulatory effects of RSV on NF-kB modulation during IBD. IL-6, interleukin 6; IL-10, interleukin 10; IL-12, interleukin 12; IL-17, interleukin 17; MHC, major histocompatibility complex; MPO, myeloperoxidase; NF-kB, nuclear factor-kB NF-kB; NO, nitric oxide; ROS, reactive oxygen species; TCR, T-cell receptor; Th0, T helper 0; Th1, T helper 1; Th2, T helper 2; Th17, T helper 17; TLR, Toll-like receptor; TNF-α, tumor factor necrosis alfa; Treg, regulatory T cell.

Figure 3 shows the main regulatory effects of RSV against NF-kB during IBD.

#### 4.5.3. 3-(4-Hydroxyphenyl)-propionic Acid

Zhang et al. [34] showed that in vitro and also in vivo, the use of 3-(4-hydroxyphenyl)-propionic acid, which is a microbial metabolite of RSV and a bioactive compound richly found in *Aloe africana*, was able to reduce the expression of NF-kB-related activation proteins and MAPK. After the treatment, the models presented elevated inflammation and OS, markedly due to an elevated expression of NF-kB and MAPK.

#### 4.5.4. Sesamol

Zhao et al. [35] performed a study with a mice model of colitis to evaluate the effects of sesamol, a bioactive constituent of sesame seeds, against the NF-kB activation during IBD. The results showed that the control mice presented elevated disease activity index (DAI), histopathological changes, gut barrier disruption before the treatment, and an increased COX-2, TNF-α, IL-6, IL-1β, iNOS, and TLR4 expressions. In turn, the treated rats presented inhibition of COX-2 mRNA, iNOS mRNA, IL-6 mRNA, IL-1β mRNA, TNF-α mRNA, and TLR4 mRNA expressions, in addition to increased p-NF-kB/NF-kB ratio.

#### 4.5.5. Kaempferol

Qu et al. [36] studied the effects of kaempferol, a flavonoid encountered in many medicinal plants, in a DSS-induced C57BL/6 mice model of colitis. The control mice presented elevated DAI scores, intestinal mucosal injury, decreased colon length, and altered gut microbiota. The use of kaempferol decreased the levels of IL-6, IL-1β, TNF-α, IL-1β mRNA, IL-6 mRNA, TNF-a mRNA, COX-2 mRNA, MCP-1 mRNA, iNOS mRNA, TLR4, NLRP3, mitogen-activated protein kinase 1 (MAPK1), MYD88 innate immune signal transduction adaptor (MyD88), and p-NF-kB-P65, as well as increased levels of Tight junction protein-1 (ZO-1), occludin, claudin-1, and IL-10.

#### 4.5.6. Astragalin

Peng et al. [37] conducted a study with a DSS-induced C57BL/6 mice model of colitis to study the effects of astragalin, a bioactive compound of *Moringa oleifera*, *Cassia alata*, and *Rosa agrestis*, against the NF-kB activation in this model of IBD. The control mice presented increased DAI scores, intestinal mucosal injury, inflammatory cell infiltration, and decreased colon length. After the treatment, the mice demonstrated downregulation of all pro-inflammatory cytokines mRNA, TLR4 mRNA and p-IκBα, p-IKKα/β, and p-p65 expressions, as well as increased expressions of ZO-1 mRNA, occludin mRNA, and Mucin 2, oligomeric mucus/gel-forming (Muc2) mRNA.

Han et al. [38] also studied astragalin treatment against IBD models. These authors used TNF-α -stimulated HCT-116 and HT-29 human colonic epithelial cells in vitro and DSS-induced C57BL/6 mice model of colitis in vivo and found that after the treatment, there was a decreased cell proliferation, TNF-α mRNA, IL-8 mRNA, IL-6 mRNA, IκBα phosphorylation, and NF-kB-DNA binding in vitro, as well as decreased TNF-α mRNA, IL-8 mRNA, IL-6 mRNA, and IκBα phosphorylation in vivo.

#### 4.5.7. Pinocembrin

Pinocembrin is a bioactive compound found in propolis, honey, wild marjoram, and the roots of ginger. Yue et al. [39] evaluated the effects of pinocembrin in vitro and in vivo study with LPS-stimulated RAW264.7 and Caco-2 cells in vitro and DSS-induced C57BL/6 mice model of colitis in vivo. Before the treatment, the cells presented elevated inflammatory stimuli measured by elevated TNF-α, COX-2, iNOS, IFN-γ, IL-6, IL-15, TLR4, p65 phosphorylation, and IκBα phosphorylation, as well as decreased NO production. After the treatment, the cells presented decreased inflammatory mediators production and NF-kB activation measured by decreased NF-kB-luciferase activity and TLR4/myeloid differentiation protein 2 (MD2)/LPS signaling activation. Additionally, the treated mice presented decreased expression of NF-kB due to decreased TLR4 mRNA, Myd88 mRNA, iNOS mRNA, COX-2 mRNA, and TNF-α mRNA expressions and p65 phosphorylation.

#### 4.5.8. Oxyberberine

Li et al. [40], in a study conducted with a DSS-induced BALB/c mice model of colitis, the effects of oxyberberine, a microbial metabolite of berberine, a bioactive compound found in several Chinese herbal medicines, against the NF-kB activation. After the treatment, the treated mice presented reduced MPO, IL-6, IL-1β, IL-17, TNF-α, IFN-γ, p65 (nucleus), p-IκBα/IκBα ratio, TLR4, and MyD88 levels, as well as decreased NF-kB-p65 translocation and IκBα phosphorylation. Additionally, the treated mice presented elevated expressions of ZO-1, zonula occludens-2 (ZO-2), occludin, junctional adhesion molecule A (JAM-A), claudin-1, and p65 (cytoplasm).

#### 4.5.9. Berberine Hydrochloride

Zhu et al. [41] investigated the effects of berberine hydrochloride, a bioactive compound encountered in *Rhizoma coptidis* and *Cortex phellodendri*, in a DSS-induced Wistar mice model of colitis. Results showed that the treatment resulted in deactivation of the NF-kB signaling expressed by decreases in IL-1 mRNA, IL-1β mRNA, IL-6 mRNA, IL-12 mRNA, TNF-α, IFN-γ mRNA, iNOS, MPO, MDA, and p-NF-kB expressions, as well as increases in IL-4 mRNA, IL-10 mRNA, p-Signal transducer and activator of transcription 3 (STAT3), ZO-1 mRNA, VCAM-1 mRNA, occludin mRNA, and claudin-1 mRNA expressions.

#### 4.5.10. Berberine

Lee et al. [42] evaluated the effects of berberine, a bioactive compound richly encountered in the rhizome of *Coptidis japonica*, in TNBS-induced C3H/HeN and C3H/HeJ mice models of colitis and found that the treated mice presented deactivation of the NF-kB due to reductions in the TLR4 expression, NF-kB phosphorylation, and nuclear translocation, as well as increases in antioxidant SOD and Catalase (CAT) and anti-inflammatory IL-10 expressions, in addition to decreases in pro-inflammatory TNF-α, IL-1β, IL-6, iNOS, and COX-2 expressions.

#### 4.5.11. Eriodictyol

Hu et al. [43] evaluated the effects of eriodictyol, a bioactive compound widely distributed in foodborne plants, in a TNBS-induced model of colitis. The treated animals demonstrated decreases in MPO activity, pro-inflammatory cytokines IL-6, IL-1β, IL 12, IL-2, and TNF-α expressions, MDA levels, p65 phosphorylation, and IκBα phosphorylation, additionally to increases in IL-10 levels, antioxidant enzymes Superoxide dismutase (SOD), CAT, and Glutathione (GSH)-Px expressions, and IκBα levels.

#### 4.5.12. Betulin

El-Sherbiny et al. [44] evaluated the effects of betulin, a bioactive compound richly found in the birch tree bark, in an acetic acid-induced Sprague Dawley model of colitis. They observed decreased lactate dehydrogenase (LDH) activity, TLR4 content, CD68 cell infiltration, IL-6, TNF-α and IL-1β content, NF-kB expression, and caspase-3 and caspase-8 activation.

#### 4.5.13. Naringin

Cao et al. [45] conducted both an in vitro and in vivo study with LPS-stimulated RAW264.7 cells and DSS-induced mice model of colitis, respectively, to evaluate the effects of naringin, a compound richly found in the grapefruit, sour orange, and citrus seed. After the treatment, the experimented animals demonstrated decreased pro-inflammatory cytokines TNF-α, IL-1β, and IL-6 expressions and NF-kB-p65 and IκB phosphorylation, p38, ERK, and c-Jun N-terminal kinase (JNK) expression levels and decreased NLRP3, ASC, and caspase-1 expression levels following increased PPARγ expression.

#### 4.5.14. 5-Hydroxy-4-methoxycanthin-6-one

Liu et al. [46] showed that 5-hydroxy-4-methoxycanthin-6-one, a bioactive compound richly found in the Ramulus et Folium picrasmae, promoted NF-kB regulation through reductions in MDA, TNF-α, IL-1β, and IL-6 expression levels, NF-kB/p65 and cluster of differentiation 3 (CD3) pro-inflammatory phenotypes, NF-kB/p65 mRNA, MYD88 and p-IκBα expression, and NF-kB/p65 nuclear translocation. The treated animals also showed increased SOD, IL-10, KKβ, and IκBα protein expression.

#### 4.5.15. Geniposide

Yang et al. [47] conducted both an in vitro and in vivo study with LPS-stimulated RAW264.7 cells in vitro and DSS-induced ICR mice model of colitis and showed that geniposide, a bioactive compound richly found in gardenia fruit, promoted NF-kB inhibition due to diminished p-NF-kBp65 and p-IκBα expression; the cells presented decreased expression levels of IL-1β, IL-6, TNF-α, and ROS but increased expression levels of SOD, nuclear factor erythroid 2–related factor 2 (Nrf2) and Heme oxygenase (HO-1).

#### 4.5.16. Sesamin

Chen et al. [48] studied a DSS-induced C57BL/6 mice model of colitis to analyze the effects of sesamin, a bioactive constituent highly found in sesame seeds. After the treatment, the experimented animals expressed decreased TNF-α, IL-1β, IL-6, p-NF-kBp65, and p-IκBα levels, NF-kB signaling and activity, and MAPK levels.

#### 4.5.17. Taxifolin

Hou et al. [49] showed that taxifolin, a natural antioxidant polyphenol with various bioactivities that is extracted from artichoke, onions, olive oil, grapes, milk thistle, citrus fruits and sorghum grain, could decrease NF-kB activation due to a reduced TNF-α, IL-1β and IL-6 expression levels, elevated Immunoglobulin A (SIgA), IL-10 and SOD expression levels and decreased p-NF-kB-p65 and p-IkBα in DSS-induced model of colitis.

#### 4.5.18. Isobavachalcone

Zhou et al. [50] studied the role of isobavachalcone, a naturally occurring chalcone first isolated from *Psoralea corylifolia*, against NF-kB activation in this model of IBD. The results showed that after treatment, the experimented mice presented reductions in the expression levels of MPO, TNF-α, IL-1β, IL-6, PGE2, NO, iNOS, and COX-2 principally due to regulation of the p-NF-kB-p65, which was decreased.

#### 4.5.19. d-Pinitol

Lin et al. [51] evaluated the effectiveness of d-pinitol, a bioactive compound richly found in soybeans, *Ceratonia siliqua* Linn and *Bruguiera gymnorrhiza*, against the activation of NF-kB in a DSS-induced BALB/c mice model of colitis. With the treatment, the mice presented decreased NF-kB signaling, which was accompanied by reduced expression levels of MPO, MDA, iNOS, COX-2, TNF-α, IFN-γ, IL-6, IL-17, and IL-1β, as well as increased expression levels of GSH, SOD, CAT, IL-10, and PPAR-γ.

#### 4.5.20. Paeoniflorin-6′-O-benzene Sulfonate

Jiang et al. [52] studied a DSS-induced mice model of colitis to evaluate the effectiveness of paeoniflorin-6′-O-benzene sulfonate, an active monomeric anti-inflammatory agent produced by chemical structural modifications of paeoniflorin, and showed decreased G-protein-coupled receptor kinase 2 (GRK2) translocation and TLR4-NF-kB-NLRP3 inflammasome signaling in macrophages.

#### 4.5.21. Thymol

Chamanara et al. [53] evaluated the roles of thymol, a dietary monoterpene phenol found in thyme species, against the activation of NF-kB in an acetic acid (AcOH) -induced model of colitis. After the treatment, the experimented group presented downregulation of p-NF-kB-p65 and decreased MPO and TNF-α expression levels.

#### 4.5.22. Tricin

Li et al. [54] conducted both an in vivo and in vitro study to evaluate the anti-inflammatory effects of the flavone tricin (5,7,4′-trihydroxy-3′,5′-dimethoxy-flavone), a bioactive isolate found mainly in wheat straw, against DSS-associated colitis in mice and LPS-induced RAW 264.7 cells. After treatment, there was a decrease in these factors, nuclear suppression of p65 phosphorylation that regulates the NF-kB pathway, and increased Treg cells.

#### 4.5.23. Aesculin

Tian et al. [55] evaluated the effects of aesculin, a constituent of *Cortex fraxini* in vivo and in vitro as anti-inflammatories by activating PPAR-γ in DSS-induced colitis in mice and LPS-induced RAW 264.7 cells. After using aesculin, there was a reduction of inflammation, of TNF-α, IL-1β and iNOS mRNA, of p-P65 in the nucleus and IkBα phosphorylation and an increase of nuclear PPARγ in both models, resulting in inhibition of the NF pathway -kB.

#### 4.5.24. Ginsenoside Rk3

Chen et al. [56] used ginsenoside Rk3, a bioactive compound extracted from the roots of *Panax notoginseng*, in high-fat diet (HFD)-induced colitis in obese mice, and observed decreased colon inflammation by reducing the expressions of MCP-1, F4/80, nicotinamide adenine dinucleotide phosphate hydrogen (NADPH), six transmembrane protein of prostate 2 (STAMP2), IL-6, IL-1β, TNF-α, TLR4, TLR4/MYD88, JNK/phosphorylation JNK, IkBα, and NF-kB itself.

#### 4.5.25. Lancemaside A

Joh et al. [57] evaluated the anti-inflammatory effects of lancemaside A, the main compound of the rhizome of *Codonopsis lanceolata*. In vitro, the treatment decreased this activation of LR4-linked NF-kB. In vivo, there was an improvement in the thickening, shortening, ulceration, edema, and inflammation of the colon, regulated by the elevation of MPO, IL-6 mRNA, IL-1β mRNA, TNF-α mRNA, TLR4 mRNA, NF-kB (p-p65), and COX-2.

#### 4.5.26. Tetramethylpyrazine

Lu et al. [58] used tetramethylpyrazine (TMP), a component of *Ligusticum wallichii* or *Ligusticum chuanxiong*, in models of colitis induced by oxazoline in vivo and LPS-stimulated Caco-2 cells in vitro. After induction, the animals showed thickening of the intestinal mucosal layer, ulceration, edema, mucus depletion, and inflammation in the colon revealed by increased nuclear NF-kB-P65, iNOS, COX-2, cellular myelocytomatosis oncogene (C-MYC), and p-cytoplasmic IKBα in both models, in addition to an increase in inflammatory cytokines (TNF-α, IL-6, IL-8, and IFN-γ) in Caco-2-cells. All these events were decreased when the models were treated or pretreated with TMP.

#### 4.5.27. Daurisoline

Zhou et al. [59] applied daurisoline (DS), a bis-benzylisoquinoline alkaloid extracted from *Menispermi* rhizoma traditionally used in China, in mice with DSS-induced colitis and in RAW 264.7 cells induced by LPS to evaluate its protective effects. In vivo, after treatment with DS, there was a reduction in diarrhea, bleeding, ulcerations, thickening, and inflammation of the colon due to the decrease in the expression of NO, COX-2, PGE2, IL-1β, MMP-9, proto-oncogene Wnt-1 (Wnt-1), β-Catenin, cyclin-D1, C-MYC, Transcription factor 4 (TCF-4), lymphoid enhancer binding factor 1 (LEF-1), glycogen synthase kinase 3 beta (GSK3β), NF-kB p-p65, and p-IκBα and increased IL-4, and IL-10. In vitro, there was a reduction in the NO, NF-kB p65, p-p65, p-IκB-α, and p65 factors and an increase in the IκB-α factor after the use of DS.

#### 4.5.28. Tetrandrine

Zhang et al. [60] showed that tetrandine, a bisbenzylisoquinoline alkaloid isolated from the Chinese plant *Stephania tetrandra*, can reduce NF-kB activity along with IL-1β and TNF-α expressions in a model of induced colitis.

#### 4.5.29. Diosgenin

Tang et al. [61] used diosgenin, a steroidal sapogenin extracted from *Trigonella foenum-graecum*. They showed that it could reduce MDA, NO, MPO, hydroxyproline, TNF-α, IL-1β, IL-6, iNOs mRNA, COX-2 mRNA, IFN-γ mRNA, Bcl-2-associated X protein (Bax), Caspase-1, NF-kB and IκBα and increase IL-10, SOD and GSH in an in vivo model of TNBS-induced colitis.

#### 4.5.30. Mangiferin

Jeong et al. [62] performed in vivo and in vitro analyses to evaluate the anti-inflammatory mechanisms of *mangiferin*, a compound found in plants such as *Mangifera indica* L., *Anemarrhena asphodeloides* and *Cyclopia intermedia*. In vitro, there was an increase in interleukin 1 receptor associated kinase 1 (IRAK1) phosphorylation and degradation, degradation of Interleukin 1 (IL-1) receptor-associated kinases (IRAK) 1, 2, and 4, increase in NF-kB activation, in transforming growth factor-β-activated kinase 1 (TAK1) phosphorylation and degradation, in IKKβ phosphorylation, in IκBα phosphorylation and degradation, in mediators inflammatory (PGE2, TNF-α expression, IL-1β expression, IL-6 expression, and COX-2), anti-inflammatory (IL-10 expression) and ROS (iNOS expression and NO). After treatment, there was a reversal of this scenario and a decrease in p65 translocation, MAPK p38 phosphorylation, ERK phosphorylation, and JNK phosphorylation. In vivo, before treatment, there was activation of IRAK1 and IKKβ and NF-kB; there was also an elevation of COX-2, iNOS, TNF-α, IL-1β, and IL-6. These conditions were suppressed by mangiferin.

#### 4.5.31. Tryptanthrin

Wang et al. [63] used tryptanthin (TRYP), a bioactive compound found in Japanese indigo, to evaluate its effects on DSS-induced colitis. Before treatment, results showed an increase in clinical activity score (CAS), tissue erosion, disappearance of crypts and goblet cells, infiltration of inflammatory cells, and elevation of cytokines TNF-α, IL-1β, and IL-6, expression of NF-kBp65 and p- STAT3 and IκBα degradation. After treatment, these findings were reduced, which demonstrates the anti-inflammatory effect of TRYP in attenuating TNF-α/NF-kB and IL-6/STAT3 pathways. There was also an increased expression of IL-10.

#### 4.5.32. l-Theanine

Zhang et al. [64] analyzed the anti-inflammatory effects of l-theanine, a tea leaf (such as in *Camellia* sinensis) compound, and glutamate derivative, on DSS-induced colitis in an in vivo model. Results showed that after induction, there was a loss of weight and colon size, augmented infiltration of inflammatory cells, and elevated release of inflammatory cytokines (TNF-α, IL-1β, and IL-6), in addition to increased COX expression -2, iNOS, p65, p-p65, p53, p-p53, and phosphorylated protein kinase B (p-Akt). After using l-theanine, this inflammatory condition was reversed due to inhibiting the NF-kB pathway.

#### 4.5.33. Koreanaside A

Kim et al. [65] worked with koreanaside A, a lignan metabolite of *Forsythia koreana*, to evaluate its effects on LPS-stimulated RAW 264.7 macrophages and DSS-induced colitis. After stimulation, the in vitro model showed an increase in the production of NO, PGE2, expression of iNOS, COX-2, IL-6, and TNF-α, transcription of activating protein-1 (AP-1) factor, NF-kB phosphorylation, and degradation of IκBα, IKKα/β, and transforming growth factor-β-activated kinase 1 (TAK1). The induced colitis decreased the weight of the mice and the size of the colon, and an increase in DAI, ulceration, loss of crypts, and infiltration of inflammatory tissue cells, and augmented the expression of COX-2, iNOS, IL-6, TNF-α, cellular proto-oncogene Fos (c-FOS), p65, Signal transducer and activator of transcription 1 (STAT1), and STAT3. After the use of koreanaside A, the inflammatory scenario was reversed in both models through the regulation of factors associated with NF-kB.

#### 4.5.34. 6-Gingerol

Sheng et al. [66] used 6-gingerol, found in ginger (*Zingiber officinale*), against DSS-induced colitis in mice. After induction, the animals showed weight loss, crypt and goblet cell destruction, granulation, hyperplasia, and inflammatory cell invasion, along with increased IL-17 and decreased IL-10. In addition, there was the deregulation of IκBα and p65, p-IκBα and p-p65 factors associated with NF-kB activation. After using 6-gingerol, the pro-inflammatory activity was reversed.

#### 4.5.35. Lycopene

Li et al. [67] evaluated the effects of lycopene in a model of DSS-induced colitis. The results showed that the animals presented weight loss, colonic size and weight gain, glandular disorder, and inflammation represented by cellular infiltration, reduction of antioxidants SOD, CAT, and GSH-Px, and increase of MDA, MPO, IFN-γ, TNF -α, IL-6 and IL-1β and expression levels of TLR4, TIR-domain-containing adapter-inducing interferon-β (TRIF), and p-NF-kB p65. After treatment, there was a decrease in the expression of pro-inflammatory mediators and levels of the TLRA4/TRIF/NF-kB pathway. An increase in the expression of antioxidant mediators and reversal of colon lesions were also observed.

#### 4.5.36. α-Mangostin

You et al. [68] used α-mangostin, the main xanthone derived from *Garcinia mangostana *L. plant. After induction of colitis by DSS, the animals presented weight loss, diarrhea, intestinal bleeding, colon shrinkage, ulceration, erosion, crypt distortion, edema, and inflammation characterized by cellular infiltration. In addition, there was an increase in MPO, phosphorylation of IKKα, IκBα, ERK1/2, stress-activated protein kinases (SAPK/JNK), and p38, and activation of NF-kB and MAPK. After treatment with α-mangostin, the histopathological findings were reversed, and the regulation of NF-kB activation factors attenuated the pro-inflammatory pathways.

#### 4.5.37. Ophiopogonin D

Wang et al. [69] used ophiopogonin D, a compound isolated from *Ophiopogon japonicus*, to analyze its effects on in vivo DSS-induced colitis and LPS-induced intestinal epithelioid cell 6 (IEC-6) treated cells. After induction of colitis, there was weight loss, diarrhea, intestinal bleeding, colon shrinkage, ulceration, erosion, edema, and inflammation marked by cellular infiltration, an elevation of MPO, phosphorylation of IKKα and IκB, ERK1/2 and SAPK/JNK, increased NF-kB and MAPK activity, and increased levels of p38 expression. In vitro, before treatment, there was an increase in cleaved (cl)-caspase3, COX-2, myosin light-chain kinase (MLCK), and iNOS, and after treatment, there was a decrease in NF-Κb-p65. After treatment, this inflammatory scenario was reversed with NF-kB control.

#### 4.5.38. Alantolactone

Ren et al. [70] showed that alantolactone, a natural sesquiterpene lactone isolated compound from *Inula helenium* and *Inula japonica*, can improve bloody diarrhea and inflammation characterized by neutrophilic infiltration and increased MPO, increased expression of pro-inflammatory cytokines (TNF-α, IFN-γ, IL-6), increased expression of pro-inflammatory mediators such as COX-2, PGE2, MCP-1, and ICAM, increased expression of oxidative factors (iNOS and NO), activation of NF-kB p65 and increased phosphorylation/degradation of IκBα. In vitro results showed a decrease in p-p65 and increased PXR transactivation and human pregnane X receptor (Hpxr) via binding to hPXR-LBD.

#### 4.5.39. Sinomenine

Xiong et al. [71] used sinomenine, a pure alkaloid extracted from the Chinese medicinal plant *Sinonium acutum*, in mice with induced colitis. After using DSS, there was a loss of weight and appetite, increase in pasty stools, shrinkage of the colon, and inflammation represented by cellular infiltration, in addition to increased expression of MyD88, NF-kBp65, TLR4, Single Ig IL-1-related receptor (SIGIRR), and increased activation of the TLR pathway /NF-kB. After using sinomenine, this inflammatory condition had micro and macroscopic characteristics reversed, in addition to a decrease in the expression of IFN-γ, IL-1β, TNF-α, IL-6, and IL-12P70.

#### 4.5.40. Convallatoxin

Li et al. [72] used *convallatoxin*, a glycoside isolated from the Chinese plant *Adonis amurensis*, to evaluate its effects in a model of UC. Both in vitro and in vivo, the colitis increased NF-kB and NF-kB-p65, COX-2, iNOS, IL-1β, IL-6, and TNF-α activity, in addition to decreasing PPARγ expression. In vivo, after treatment, there was a reduction in the activation of NF-kB-p65 and NF-kB mRNA, in the expression of pro-inflammatory cytokines (IL-1β, IL-6, TNF-α) and the phosphorylation of p-IκBα, in addition to increased expression of PPARγ and improvement in colon size, colon and spleen weight, decreased cellular inflammatory infiltration, ulceration, necrosis, congestion, and edema. In vitro, after treatment, there was a decrease in NF-kB p65, NF-kB mRNA, IL-1β mRNA, IL-6 mRNA, TNF-α mRNA, and p-IκBα, and an increase in PPARγ mRNA, PPARγ siRNA, and PPARγ.

#### 4.5.41. Fisetin

Sahu et al. [73] used fisetin, a flavonoid found in various fruits and vegetables (persimmons, mangoes, grapes, apples, strawberries, peaches, cucumbers, onions, and tomatoes), in DSS-induced colitis in mice and LPS-induced cells. In vitro, there was an increase in nitrites, inflammatory cytokines (TNF-α, IL-1β, and IL-6), COX-2, iNOS, NF-kB-p65 nuclear translocation, IkBα phosphorylation, and degradation. After treatment, there was a reversal of this scenario. In vivo, before treatment, there was a decrease in weight and size of the colon, loss of crypts and goblet cells, and inflammation marked by infiltration, elevation of MPO, TNF-α, IL-1β, IL-6, nitrites, COX-2, iNOS, nuclear NF-kB (p65), phosphorylation of IκBα (p-IκBα/IκBα), NF-kB (p65)-DNA binding activity, p-p38/p38, p-ERK/ERK, Akt phosphorylation, and thiobarbituric acid reactive substances (TBARS), and GSH reduction. After treatment, there was a reversal of the histological and inflammatory pattern; however, p-ERK/ERK remained high.

#### 4.5.42. Genipin

Li et al. [74] studied genipin, one of the major components in gardenia fruit (*Gardenia jasminoides*), against the activation of the NF-kB in a DSS-induced C57BL/6 mice model of colitis. The control mice presented decreased body weight, elevated intestinal epithelial destruction, massive crypt abscesses, and a reduced number of goblet cells. The mice also presented elevated MPO, MDA, TNF-α, IL-1β, and NF-kB signaling, as well as decreased Nrf2 and HO-1. After the treatment, the mice had the NF-kB signaling controlled and presented decreased MPO, MDA, TNF-α, and IL-1β and elevated HO-1 expressions.

#### 4.5.43. Piperine

Guo et al. [75] studied the effects of piperine, a bioactive constituent derived from the *Piper nigrum* plant, in a TNBS-induced model of colitis. The control mice presented diminished body weight and augmented colon weight-to-length ratio and ulceration. The animals also presented elevated oxide-nitrosative stress, iNOS, TNF-α, IL-1β, IFN-γ, COX-2 mRNA, leukotriene B4 (LTB4), IkBα, NF-kB signaling, and caspase-1, as well as decreased occludin, claudin-1, zonula occludens-1, and IL-10. With the treatment, the experimented mice presented decreased oxide-nitrosative stress, iNOS, TNF-α, IL-1β, IFN-γ, COX-2 mRNA, LTB4, caspase-1, and IkBα expression levels, and NF-kB signaling, as well as increased occludin, claudin-1, zonula occludens-1, and IL-10 expression levels.

#### 4.5.44. Ligustilide

Huang et al. [76] conducted an in vivo study to investigate the actions of ligustilide, a bioactive compound richly found in *Angelica acutiloba* and *Cnidium officinale*, in activating NF-kB in a DSS-induced C57BL/6 mice model of colitis. The controls exhibited decreased body weight and colon length and increased diarrhea, rectal bleeding, ulceration, and inflammatory cell infiltration. In addition, the controls exhibited increased MPO, iNOS, TNF-α, IL-1β, IL-6, IL-12, macrophage inflammatory protein 1 α (MIP-1α), and IL-17 with a decreased expression of PPARγ and augmented expression of NF-kB-p65. In turn, the treated animals had lower expression of NF-kB-p65 with a higher expression and signaling of PPARγ, as well as decreased expression levels of MPO, iNOS, TNF-α, IL-1β, IL-6, IL-12, MIP-1α, and IL-17.

#### 4.5.45. Evodiamine

Shen et al. [77] studied the effects of evodiamine, a bioactive compound extracted from the traditional Chinese medicine *Evodia rutaecarpa*, in a mice model of colitis. After the treatment, the experimented mice expressed decreased levels of MPO, TNF-α, IL-1β, IL-6, p-NF-kB p65, p-IkB, NLRP3, ASC, and caspase-1, as well as increased levels of ZO-1 and occludin.

#### 4.5.46. Chrysin

Dou et al. [78] studied the effects of chrysin, a flavonoid found in many plant extracts, honey, and propolis, in a TNBS-induced C57BL/6 mice model of colitis. The control mice presented weight loss, diarrhea, fecal bleeding, crypt distortion, and inflammatory exudate in the intestine. Additionally, the controls expressed elevated p-65, IkBα phosphorylation and degradation, NF-kB nuclear translocation, iNOS mRNA, ICAM-1 mRNA, MCP-1 mRNA, COX-2 mRNA, TNF-α mRNA, IL-6 mRNA, and MPO. The treated mice showed reversed inflammatory pattern through decreased p-65, IkBα phosphorylation and degradation, and NF-kB nuclear translocation, as well as by diminished expression of iNOS mRNA, ICAM-1 mRNA, MCP-1 mRNA, COX-2 mRNA, TNF-α mRNA, IL-6 mRNA, and MPO.

#### 4.5.47. Wogonoside

Sun et al. [79] studied the effects of wogonoside, a glucuronide metabolite of the bioactive flavonoid wogonin (found in *Scutellaria baicalensis*), in an induced colitis model. In vitro, after treatment, there was a reduction in IL-1β mRNA, TNF-α mRNA, IL-6 mRNA, IkBa phosphorylation, phosphorylation of p65, NF-kB DNA binding activity, NLRP3 mRNA and pro-caspase-1 mRNA. In vivo, the histological lesions were reversed after treatment, and the levels of NF-kB, NF-kB p65, NF-kB DNA binding activity, IkBa phosphorylation, p65, and p65 phosphorylation were reduced.

#### 4.5.48. Oxymatrine

Li et al. [80] used oxymatrine, a quinolizidine alkaloid extracted from *Sophora flavescens*, to evaluate its mechanism in TNBS-induced colitis. After treatment, there was a reduction in IL-1β mRNA, TNF-α mRNA, and IL-6 mRNA, in addition to a reduction in NF-kB activation and decreases in toll-like receptor 9 (TLR9) and Myd88 expression in the TLR9/Myd88/NF-kB pathway.

#### 4.5.49. Epicatechin

Zhang et al. [81] used epicatechin, a polyphenolic and oligomeric compound belonging to the proanthocyanidins found in raspberries, grapes, apples, and cocoa beans, to evaluate its effects on DSS-induced colitis in mice. With treatment, there was a reversal of histological lesions and reduction of NO, cytokines (TNF-α and IL-6), expression of antioxidant species (SOD, GSH-Px, and CAT), and a reduction of MDA.

#### 4.5.50. Thymoquinone

Venkataraman et al. [82] used thymoquinone, a bioactive molecule from the quinone-monoterpene family present in the volatile oil of *Nigella sativa *L. seeds, in a model of induced colitis. After treatment, there was a reduction in the expression of chemokine (C-X-C motif) ligand 1 (CXCL-1), IL-8, and COX-2, and an increased expression of PPAR-γ. In vivo, after treatment, there was a reversal of histological damage and a decrease in IL-6 mRNA, IL-1β mRNA, TNF-α mRNA, p-ERK, p-JNK, p-p38, and phospho-NF-kB protein, in addition to PPAR elevation.

#### 4.5.51. Fraxinellone

Wu et al. [83] investigated the effects of fraxinellone, a naturally occurring lactone (*Dictamnus dasycarpus*), on the NF-kB pathway and NLRP3 inflammasome in a model of colitis. In vitro, after treatment, there was a reduction in IL-1β and IL-18 expression, phosphorylation of IKKα/β, IκBα, p65 phosphorylation, p65, Caspase-1 activation, and NLRP3 inflammasome. After induction of colitis by DSS, there was weight loss, diarrhea, fecal bleeding, increased mortality, ulcers, loss of colon, and inflammation configured by cellular infiltration, the elevation of pro-inflammatory cytokines (IL-1β, IL-18, TNF-α, and IL-6) and NO. However, treatment with fraxinellone reversed the morphological lesions and reduced MPO, inflammatory cytokines, and expression of VCAM1, iNOS, and COX-2, in addition to increasing glutathione.

#### 4.5.52. Artesunate

In a colitis model, artesunate, a synthetic artemisinin derivative isolated from the artemisia plant, was used by Chen et al. [84]. In vitro, before treatment, the inflammation scenario was marked by increased cell infiltration, TNF- α, IL-8, IFN-γ, p-NF-kB, p-p38, Bax, and caspase-9 levels, and reduction of Bcl-2 and Cell Counting Kit-8 (CCK-8). After treatment, there was a reversal of this scenario. After induction of colitis in rats, there was a drop in hemoglobin, colon, and elevation of DAI, cell destruction, and inflammation marked by cell infiltration, the elevation of MPO and pro-inflammatory cytokines (TNF-α, IL-8, and IFN-γ), TLR4, p-NF-kB, p-p38, Bax, caspase-9, in addition to a decrease in Bcl-2. Artesunate treatment reversed the histological findings, decreased inflammatory cytokines, TLR4, p-NF-kB, p-p38, Bax, and caspase-9, and increased Bcl-2 expression.

#### 4.5.53. Aesculetin

Wang et al. [85] considered aesculetin, a coumarin derivative extracted from the bark of *Fraxinus rhynchopylla*, to investigate its effects in activating the NF-kB pathway and MAPKs in vivo and in vitro model of colitis. In vitro, before treatment, there was an increase in NO, iNOS expression, p–NF–κB-P65 expression, NF-kB P65 nuclear translocation, p38 phosphorylation, JNK phosphorylation, ERK phosphorylation, and NLRP3 expression. After treatment, there was a reversal of this situation. DSS in mice presented a decrease in colon and colon weight, an increase in DAI, and inflammation marked by cell infiltration, MPO, the elevation of cytokines (TNF-α and IL-6), and p–NF–κB-P65 expression, nuclear translocation of NF-kB P65, p38, JNK, and ERK phosphorylation. These inflammatory aspects were reverted when the animals were treated with aesculetin.

#### 4.5.54. Euphol

Dutra et al. [86] used euphol, obtained from *Euphorbia tirucalli*, to evaluate its anti-inflammatory activity in colitis models. Colitis induced by DSS and TNBS caused a hemorrhage in the colonic lumen, weight loss, bloody diarrhea, hyperemia, necrosis, reduction of crypts and goblet cells and presence of inflammation marked by cell infiltration, increased MPO, increase in pro-inflammatory cytokines (IL-1β, CXCL1, MIP-2, MCP-1, TNF-α, and IL-6), increase in expressions of nitric oxide synthase 2 (inducible) (NOS2), vascular endothelial growth factor (VEGF), antigen KI-67 (Ki67), ICAM-1, VCAM-1 and Lymphocyte function-associated antigen 1 (LFA-1) and increased phosphorylation of NF-kB-p65. After treatment with euphol, this inflammatory condition and the histopathological lesions were reversed, mainly due to the anti-NF-kB activity.

#### 4.5.55. Nobiletin

Xiong et al. [87] used nobiletin, a citrus polymethylated flavonoid extracted from citrus peel, to evaluate its properties in colitis models. In vitro, before treatment, there was an increase in Akt, MLCK mRNA, MLCK protein, and NF-kB p65 protein expression. After treatment, there was a reversal of this scenario. After colitis induction, the animals showed loss of weight and food intake, increased colon-weight-to-length ratio, and inflammation characterized by elevation of MPO, pro-inflammatory cytokines (IL-1β, IL-6, and TNF-α), NO, PGE2, iNOS expression, COX-2 expression, and the MLCK, NF-kB and NF-kB-p65 protein expression, phosphoinositide 3-kinase (PI3K) and Akt pathways. After treatment, there was a decrease in these inflammatory patterns due to the interference of nobiletin in the NF-kB activation pathways and improvement in the histopathological condition.

#### 4.5.56. Galangin

Gerges et al. [88] used galangin, a flavonol present in different types of propolis, such as Egyptian propolis and *Alpinia officinarum*, to evaluate its therapeutic potential in an in vivo model of colitis. The results showed that after the induction of colitis by DSS in mice, the animals showed an increase in ulceration, necrosis, and intestinal inflammation marked by cell infiltration, increased expression of TLR4, and High mobility group box 1 (HMGB1), MDA, nuclear NF-kB-p65 and pro-inflammatory cytokines (TNF-α and IL-6), in addition to the reduction of antioxidant species (GSH). However, treatment with galangin reversed this inflammatory condition by inhibiting NF-kB activation, interfering with NF-kB-p65 translocation.

## 5. Conclusions

In summary, IBD are very common diseases associated primarily with a massive activation of the NF-kB pathways even during their first stages, positively affecting the disease progression. Nowadays, the clinical goal of regulation of NF-kB during IBD by inhibiting its activation is a field of study. However, many side effects derived from synthetic anti-inflammatory drugs and pharmacies can damage other organs and systems than the gastrointestinal. As an important source of innovative treatments against IBD-related NF-kB activation, phytochemicals with biological activities regulating NF-kB have been explored and investigated. They are derived from medicinal plants and do not lead to serious adverse effects. The modulatory mechanisms involved in NF-kB regulation by phytochemicals result in decreased levels of TNF-α, IL-1β, IL-6, IFN-γ, and COX-2, and augmented occludin, claudin-1, zonula occludens-1, and IL-10 expression. Thus, further research should focus on the efficacy and safety of phytochemicals and other natural regulators of NF-kB on IBD.
